# Placebo and nocebo effects in gambling disorder pharmacological trials: a meta-analysis

**DOI:** 10.1017/neu.2024.52

**Published:** 2024-11-20

**Authors:** Konstantinos Ioannidis, Nathan T.M. Huneke, Jeremy E. Solly, Guilherme Fusetto Veronesi, Charidimos Tzagarakis, Valeria Parlatini, Samuel J. Westwood, Cinzia Del Giovane, David S. Baldwin, Jon E. Grant, Samuele Cortese, Samuel R. Chamberlain

**Affiliations:** 1 Department of Psychiatry, Clinical and Experimental Sciences, Faculty of Medicine, University of Southampton, UK; 2 Hampshire and Isle of Wight Healthcare NHS Foundation Trust, Southampton, UK; 3 Cambridgeshire and Peterborough NHS Foundation Trust, Cambridge, UK; 4 Department of Psychiatry, University of Cambridge, UK; 5 Department of Psychiatry, School of Medicine, University of Crete, Iraklion, Greece; 6 Organization Against Drugs (OKANA), Athens, Greece; 7 Department of Neuroscience, University of Minnesota, Minneapolis, MN, USA; 8 Department of Child and Adolescent Psychiatry, Institute of Psychiatry, Psychology and Neuroscience, King’s College London, London, UK; 9 Department of Forensic and Neurodevelopmental Sciences, Institute of Psychiatry, Psychology and Neuroscience, King’s College London, London, UK; 10 Centre for Innovation in Mental Health, School of Psychology, Faculty of Environmental and Life Sciences, University of Southampton, Southampton, UK; 11 Department of Psychology, Institute of Psychiatry, Psychology and Neuroscience, King’s College London, London, UK; 12 Department of Medical and Surgical Sciences for Children and Adults, University of Modena and Reggio Emilia, Modena, Italy; 13 Institute of Primary Health Care (BIHAM), University of Bern, Bern, Switzerland; 14 Department of Psychiatry and Behavioral Neuroscience, University of Chicago, Chicago, IL, USA; 15 Clinical and Experimental Sciences (CNS and Psychiatry), Faculty of Medicine, University of Southampton, Southampton, UK; 16 DiMePRe-J-Department of Precision and Rigenerative Medicine-Jonic Area, University of Bari ‘Aldo Moro’, Bari, Italy; 17 Department of Child and Adolescent Psychiatry, New York University Grossman School of Medicine, New York, USA

**Keywords:** Gambling, placebo, meta-analysis, pharmacotherapy, treatment

## Abstract

**Background:**

Placebo and nocebo effects are widely reported across psychiatric conditions, yet have seldom been examined in the context of gambling disorder. Through meta-analysis, we examined placebo effects, their moderating factors, and nocebo effects, from available randomised, controlled pharmacological clinical trials in gambling disorder.

**Methods:**

We searched, up to 19 February 2024, a broad range of databases, for double-blind randomised controlled trials (RCTs) of medications for gambling disorder. Outcomes were gambling symptom severity and quality of life (for efficacy), and drop outs due to medication side effects in the placebo arms.

**Results:**

We included 16 RCTs (*n* = 833) in the meta-analysis. The overall effect size for gambling severity reduction in the placebo arms was 1.18 (95%CI 0.91–1.46) and for quality of life improvement was 0.63 (0.42-0.83). Medication class, study sponsorship, trial duration, baseline severity of gambling and publication year significantly moderated effect sizes for at least some of these outcome measures. Author conflict of interest, placebo run-in, gender split, severity scale choice, age of participants or unbalanced randomisation did not moderate effect sizes. Nocebo effects leading to drop out from the trial were observed in 6% of participants in trials involving antipsychotics, while this was less for other medication types.

**Conclusion:**

Placebo effects in trials of pharmacological treatment of gambling disorder are large, and there are several moderators of this effect. Nocebo effects were measureable and may be influenced by medication class being studied. Practical implications of these new findings for the field are discussed, along with recommendations for future clinical trials.

Significant outcomes

- Placebo effects in trials of pharmacological treatment of gambling disorder are large.
- Medication class, study sponsorship, trial duration, baseline severity of gambling and publication year significantly moderated the placebo effect.
- Nocebo effects were measurable and may be influenced by medication class being studied.

Significant limitations

- The number of RCTs available for the pharmacological management of gambling disorder is relatively limited to date, as compared to other areas of mental ill health.
- The presence or not of comorbidities was not comprehensively assessed in those RCTs, which limited the possibility of those being identified as moderators.
- Meta-regression was not possible for blinding integrity, as this was not measured in those RCTs.

## Introduction

Gambling disorder is a complex mental disorder characterised by persistent and recurrent gambling despite the evidence of negative consequences. It is classified as a behavioural addiction in the International classification of Disease 11^th^ Edition (ICD-11) (ICD-11, 2021) and as a Substance-Related and Addictive Disorder in the Diagnostic and Statistical Manual 5^th^ Edition, text revised (DSM-5-TR) (American Psychiatric Association, 2022). Gambling Disorder has a substantial impact on those affected, as well as those around them. For example, it can lead to interpersonal conflict, serious financial problems, homelessness, bankruptcy, and elevated risk of suicide (The Lancet Public Health, 2021; Wardle & McManus, 2021; Ioannidis & Bowden-Jones, 2023) with substantial public health implications worldwide (The Lancet Public Health, 2021).

Psychological treatments (i.e. mainly in the form of gambling focused cognitive-behavioural therapy, CBT, in its many variants) comprise the current mainstay approach for the treatment of gambling disorder. Pharmacological treatments are also available, with opioid receptor antagonists (nalmefene, naltrexone) currently having the best evidence (Ioannidis *et al*., 2024), although no pharmacological treatment is licensed for this indication (as is the case for many neglected mental disorders). In the emerging body of randomised clinical trials (RCTs) for gambling disorder, most active medication treatments have been compared against placebo treatment. Placebo and nocebo effects may have occurred in these trials. Placebo and nocebo are effects of patients’ positive and negative expectations relevant to an anticipated intervention; they are pertinent influencers in pharmacological trials in many areas of medicine (Colloca & Barsky, 2020). While under-studied (Huneke, *et al*., 2020), it is likely that placebo and nocebo effects can occur due to a variety of mechanisms (e.g. expectations, interactions with the study team, therapeutic milieu, the choice of self-report versus objective measures (Huneke *et al*., 2024)), and particular neurobiological pathways (Flaten, *et al*., 1999; Barsky, *et al*., 2002; Wager & Atlas, 2015; Ashar, *et al*., 2017). It is argued that, particularly in psychiatry, the predictors and moderators of the placebo response are multiple, diverse and still to be discovered (Weimer, *et al*., 2015).

It is known from empirical evidence (Huneke *et al*., 2024) that the placebo effect has variable magnitude, which differs by disorder (e.g. RCTs of medication treatments for generalised anxiety disorder or depression have reported much larger placebo effects as compared to other disorders such as obsessive-compulsive disorder [OCD] or schizophrenia spectrum conditions). At the same time there has not been any comprehensive and in depth investigation of the size and moderating parameters for the placebo effects in gambling pharmacological RCTs.

To our knowledge, only two previous studies have explored potential moderators of placebo response in pharmacological trials for gambling disorder. First, in a previous synthesis of data from *n* = 152 patients (not systematically collected), placebo ‘responders’ (defined using a cut-off of 35% reduction in symptom severity on the Gambling Symptom Assessment Scale) remained in treatment for significantly longer, were more likely to report ‘enjoyment’ as a trigger for gambling, and were less likely to state that ‘boredom’ or ‘loneliness’ triggered their gambling, compared with ‘non-responders’ (J. Grant & Chamberlain, 2017). Second, in a previous meta-analysis of individual patient data from a selected subset of RCTs (6 studies, n placebo = 67) of the pharmacological management of gambling disorder, decreased baseline symptoms of anxiety, increased baseline symptoms of depression, and non-Caucasian ethnicity were associated with larger placebo response (Huneke, *et al*., 2021). Intriguingly, these moderators differed from moderators associated with larger treatment response. Although these studies have identified possible important moderators of placebo responses in gambling disorder, they are based on an incomplete analysis exploring only a subset of the available data.

In the present study we sought to synthesise data from all the available literature to address the following key questions related to the placebo effect in pharmacological RCTs of gambling disorder: 1) what is the magnitude of placebo effect in available trials and does it differ across outcome measures 2) what factors moderate the placebo effect, 3) what is the magnitude of nocebo effect and does this differ depending on the medication class being examined?

## Material and methods

For the identification of available RCTs for the pharmacological management of gambling disorder, the study followed methods described in the pre-registered protocol published on the PROSPERO International prospective register of systematic reviews [Registration number: CRD42022329520 Available from: https://www.crd.york.ac.uk/prospero/display_record.php?RecordID=329520]. This study reporting followed the PRISMA guidelines (Shamseer *et al*., 2015).

### Search strategy

We searched up to 19 February 2024, a broad range of databases, including MEDLINE, EMBASE, PsycINFO, PubMed, CINAHL, AMED, and the Cochrane Database of Systematic Reviews, ERIC and Web of Science (including Science Citation Index Expanded (SCI-EXPANDED), Social Science Citation Index, Conference Proceedings Citation Index-Science (CPCI-S) and Conference Proceedings Citation Index-Social Science and Humanities (CPCI-SSH)) via Web of Knowledge and the WHO International Trials Registry Platform (including ClinicalTrials.gov). The search strings used and full list of electronic databases and clinical trial registries in which the search was conducted are available in the previous publication (Ioannidis, *et al*., 2024).

### Eligibility criteria

We included published or unpublished RCTs comparing an active medication versus placebo, for the treatment of Gambling Disorder/Pathological Gambling. Trials with a cross-over design were included if data from the pre cross-over phase were available, to avoid carry-over effects. We included only studies of adults (>18yrs) with a primary DSM (III onwards) or ICD (9 onwards) diagnosis of Gambling Disorder/Pathological Gambling.

### Data extraction and outcomes

Details on data extraction were described in previous work and are available online (Ioannidis, *et al*., 2024). Additional information about the ascertainment of placebo control processes were collected in this dataset, including 1) the presence of a placebo run-in process (‘Yes/No/Unclear’), use of independent investigators (not involved in clinical care) to assess side effects (‘Yes/No/Unclear’), prevention of side effects (‘Yes/No/Unclear’) and assessment of blinding success (‘Yes/No/Unclear’), 2) full details of nocebo effects (specific side effects produced by placebo, attributed to medication effects) 3) the presence of industry-related influences, including the presence of industry sponsorship (‘Yes/No/Unclear’) and the presence of declared industry-related conflict of interest from the manuscript authors in each respective publication (‘Yes/No/Unclear’).

### Clinical measures of efficacy

The primary efficacy outcome was gambling symptom severity measured by well-established and validated instruments, namely the Yale-Brown Obsessive-Compulsive Scale adapted for Pathological Gambling (PG-YBOCS), (Pallanti, *et al.*, 2005) the Gambling Symptom Assessment Scale (G-SAS), (Kim, *et al*., 2001) and the Clinical Global Impression-Improvement scale (CGI-I) (Busner & Targum, 2007). If a study reported results from multiple scales, we used the following hierarchy in the choice of the scale: PG-YBOCS as first preference; G-SAS as next preference, CGI-I as third preference. This was done to prioritise structured clinical instruments against unstructured or self-report instruments. The secondary efficacy outcome was the improvement in quality of life and functioning as measured by validated instruments including but not limited to the Sheehan Disability Scale (Sheehan, *et al*., 1996) and other quality of life metrics. Further information about the selection of severity metrics are presented in previous work (Ioannidis, *et al*., 2024).

### Nocebo outcomes

Nocebo effects were calculated by the percentage of drop outs from perceived ‘medication side effects’ from the placebo arms, in the RCTs.

### Data synthesis

Data were analysed using statistical software R version 4.2.1. Meta-analysis was performed using packages of ‘robumeta’ and ‘metafor’ (Fisher, *et al*., 2017; Viechtbauer, 2020). The R code used for this analysis is shared in the supplement. We performed analyses for gambling severity, using all types of outcomes, using a hierarchical approach described above, and then for each type of clinical severity outcome separately. We also performed a meta-analysis for quality of life outcomes; all the above were performed separately for the placebo and the treatment arms. We calculated the within-group Standardised Mean Change using Change score standardisation (Gibbons, *et al*., 1993) (SMCC), in active medication and placebo arms separately, to measure the efficacy outcomes. For the calculations of SMCC, we first imputed a correlation of 0.50 between baseline and end-of-treatment within groups. We performed sensitivity analyses with correlations of 0.25 and 0.75 to ascertain any impact on the analyses. The measure of effect for nocebo was the dropout rate due to medication side effects (or attributed to medication side effects in the placebo arm, i.e. nocebo effects), expressed as percentage of drop out, in the placebo arms. Study arms randomising the same compound at different dose were merged into a single arm in line with the recommendations in the Cochrane handbook (The Cochrane Collaboration, 2011). We meta-analysed these outcomes through a random-effects model in all cases to provide a more generalisable model estimate. We ascertained heterogeneity by calculating the Q-statistic, which is the ratio of observed variation to within-study variance, formulated in a null hypothesis test (the null hypothesis here being that all individual studies measure/examine the same effect). The test indicates how much of the overall heterogeneity can be attributed to true between-studies variation. We also assessed the heterogeneity within each comparison visually by considering the forest plot, and quantitatively with the I^2^ statistic and the τ^2^. (Higgins *et al*., 2019). Moderator meta-regression analysis was conducted considering the following regressors: year of publication, medication class, presence of company sponsor, duration of the study, gambling severity scale choice, mean age of participants, percentage of gender split, baseline gambling severity scores, presence of declared conflict of interest in the authors and study design with unbalanced randomisation and placebo run-in phase. We calculated the correlation between the placebo effect sizes and the respective effect sizes for the treatment arms in each meta-analysis.

### Publication bias assessment

We examined publication bias with the use of funnel plots (visual/graphic inspection for asymmetry), as well as with regression tests for funnel plot asymmetry. Where appropriate we used the trim and fill method to provide an updated effect size estimate.

### Risk of bias assessment

For within-study bias, and to assess the methodological quality of each individual RCT included in our meta-analysis we used the Cochrane Risk of Bias tool version 2 (RoB2) (Cochrane, 2023).

## Results

**Figure 1.**
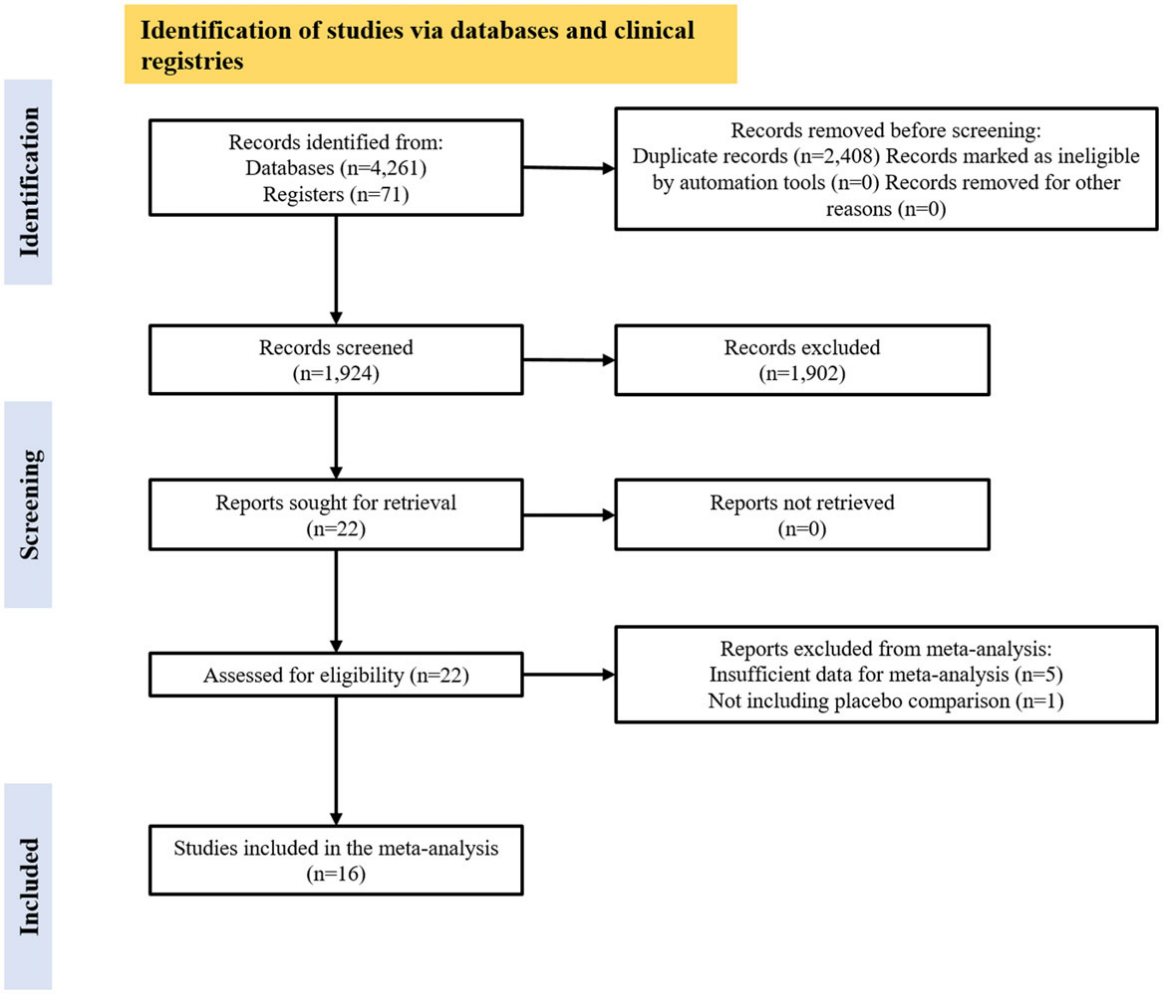
PRISMA flowchart. Comparisons included in the meta-analysis comprised eleven different medications grouped in six classes: three opioid receptor antagonists (naltrexone, nalmefene, naloxone); two selective serotonin reuptake inhibitors (SSRIs – paroxetine and fluvoxamine); two mood stabilisers (topiramate, lithium); one norepinephrine–dopamine reuptake inhibitor (NDRI, bupropion); one antipsychotic (olanzapine); and two supplements (N-acetyl-cysteine, silymarin).

The search yielded 4261 references from electronic databases and 71 hits from clinical trial registries. A final set of 16 eligible RCTs were selected for inclusion in the meta-analyses. Full details about the search results are presented in the PRISMA flowchart (Fig. 1). Randomised participants across the included RCTs were ∼ 47% males (394/833), and their ages ranged from 36.2 to 51.5 years (Mean = 43.30; standard deviation SD = 3.38). Each of the 16 RCTs included in the meta-analysis (total participants: 833) contributed to one pairwise comparison (active treatment vs. placebo), totalling 16 pairwise comparisons across studies (16 for gambling severity, five for quality of life).

### Placebo response

**Figure 2.**
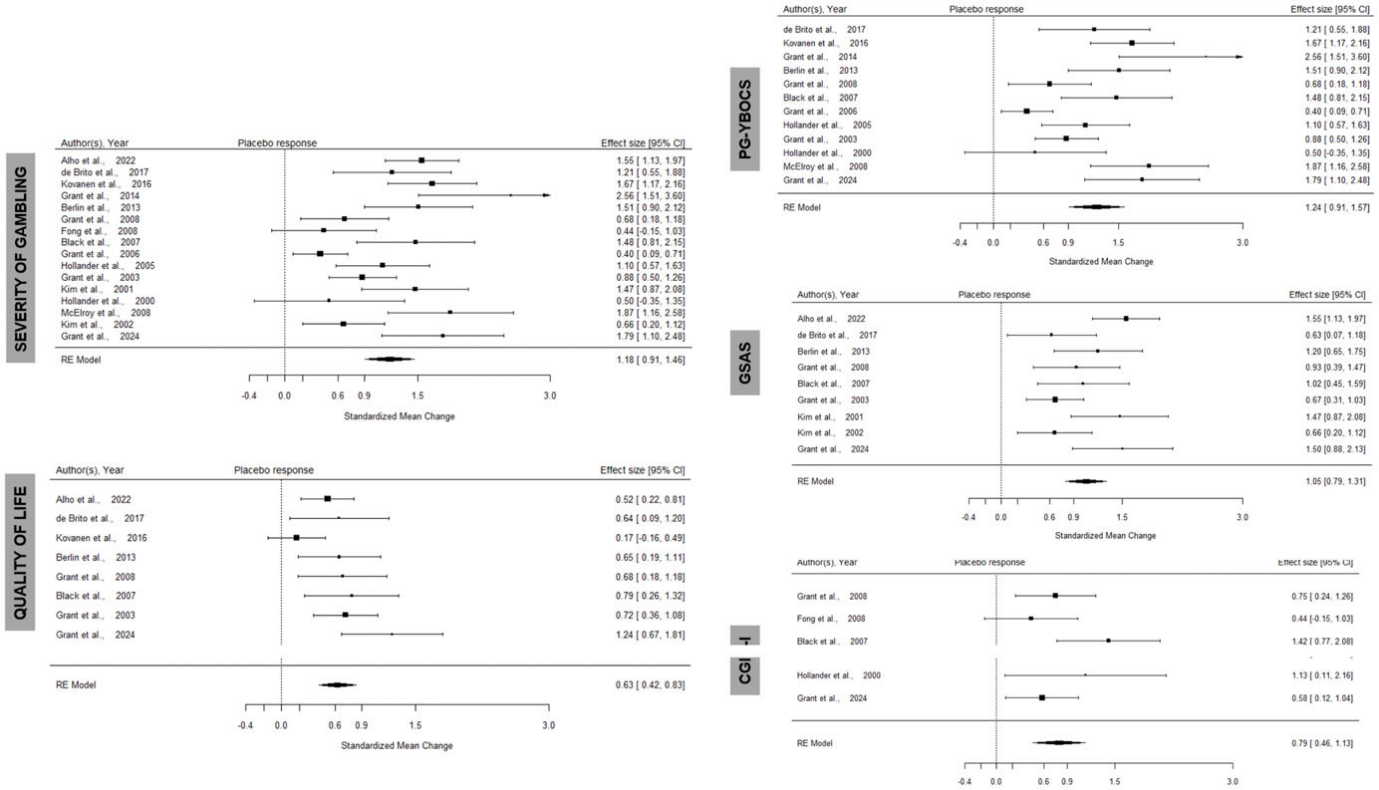
Forest plots meta-analysis of placebo effect in gambling severity (all studies, top left, per specific scale on the right hand side) in RCTs of pharmacological management of gambling disorder; ‘higher response’ indicates improvement in gambling symptom severity compared to baseline; placebo effect for quality of life outcomes (bottom left) in RCTs of pharmacological management of gambling disorder; ‘higher response’ indicates improvement in quality of life compared to baseline.

Placebo response (baseline to end-of-treatment) for the severity of gambling (all scales) was SMCC = 1.18 (95%CI 0.91-1.46), whereas for the specific gambling severity scales the effect sizes were 1.24 (95%CI 0.91-1.57) for PG-YBOCS, 1.05 (95%CI 0.79-1.31) for GSAS and 0.79 (95%CI 0.46-1.13) for CGI-I. Quality of life effect sizes were SMCC = 0.63 (95%CI 0.42-0.83) (see Fig. 2). For reference, under active treatment arms, overall gambling severity effect size was SMCC = 1.49 (95%CI 1.18-1.80), whereas quality of life effect was *g* = 0.69 (95%CI 0.55-0.84). Full details are available in the online supplement (paragraph §S1).

### Heterogeneity within placebo arms

We identified moderate to high variance from heterogeneity measures within the analysed studies of the placebo arms, which was particularly prominent in the studies that used the PG-YBOCS instrument (see Table 1). Heterogeneity in the treatment arms followed a similar pattern (paragraph §S2).

**Table 1.** Heterogeneity measures

	tau ^ 2 (estimated amount of total heterogeneity):	tau (square root of estimated tau ^ 2 value):	I ^ 2 (total heterogeneity / total variability):	H ^ 2 (total variability / sampling variability):	Q Test for Heterogeneity:
**Placebo gambling severity**	0.2243 (SE = 0.1139)	0.4736	75.50%	4.08	Q(df = 15) = 63.4507, p-val < 0.0001
**Placebo QOL**	0.0398 (SE = 0.0468)	0.1995	46.42%	1.87	Q(df = 7) = 12.9432, p-val = 0.0735
**Placebo PG-YBOCS**	0.2456 (SE = 0.1454)	0.4955	76.16%	4.19	Q(df = 11) = 49.1562, p-val < 0.0001
**Placebo GSAS**	0.0862 (SE = 0.0770)	0.2937	57.03%	2.33	Q(df = 8) = 19.2183, p-val = 0.0137
**Placebo CGI-I**	0.0498 (SE = 0.1009)	0.2232	34.86%	1.54	Q(df = 4) = 6.1393, p-val = 0.1890

PG-YBOCS = Yale-Brown Obsessive-Compulsive Scale adapted for Pathological Gambling, GSAS = Gambling Symptom Assessment Scale, CGI-I = Clinical Global Impression-Improvement scale; SE = standard error; df = degrees of freedom.

### Publication bias assessment

**Figure 3.**
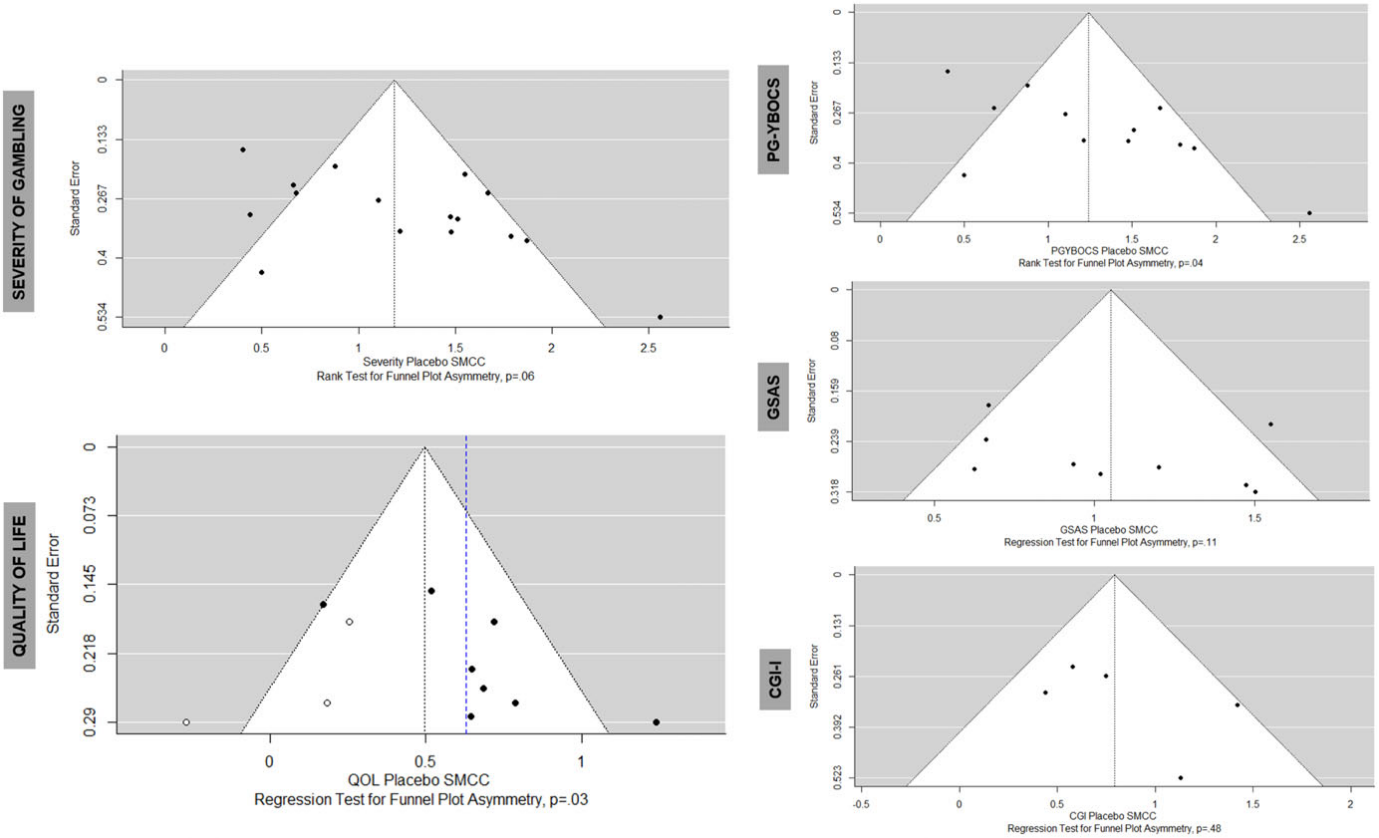
Funnel plots. Funnel plots with regression for funnel plot asymmetry p-values. PG-YBOCS = yale-brown obsessive compulsive scale adapted for pathological gambling, GSAS = gambling symptom assessment scale, CGI-I = clinical global impression-improvement scale. Publication bias identified in the quality of life analysis and the trim and fill method was used to provide a new effect size estimate.

Publication bias was identified graphically and statistically in the case of quality of life, in which the trim and fill method suggested a more conservative placebo effect estimate (0.50 vs. 0.65), which was not present in the treatment arms (Fig. 3). Publication bias assessment for treatment arms is presented in the supplement (paragraph §S3).

### Meta-regression analysis

Meta-regression results are presented in detail in Table 2. For gambling severity, recency of publication, supplement medication class and the absence of company sponsorship were associated with higher placebo effect sizes. For gambling severity using PG-YBOCS only, recency of publication, supplement medication class, as well as higher severity of baseline symptoms were associated with higher placebo effect sizes. For gambling severity using GSAS only, supplement and opiate receptor antagonist medication classes and the absence of company sponsorship were associated with higher placebo effect sizes. For CGI, antipsychotics and supplement medication classes were associated with lower placebo response. In the moderation analysis of quality of life, shorter trial duration was associated with larger placebo response, whereas longer duration was associated with lower response. Lower baseline of symptoms was associated with lower placebo response. No other parameters were statistically associated with moderating effect sizes in relation to placebo response on symptom severity or quality of life. Full results for the treatment arms are presented in the supplementary materials (paragraph §S4).

**Table 2.** Meta-regression – PLACEBO effects

	Publication Year	Medication type (class)	Sponsored study (Y/N)	Participant age (mean)	% gender	Duration of study	Severity scale choice	Unbalanced randomisation	Baseline severity	Author COI (Y/N)	Placebo run-in 1 week
**Severity (all measures)**	**Recent higher E.S. † / ††	*supplement higher E.S. † / ††	.No sponsor higher E.S. † / ††	n.s. † / ††	n.s. † / ††	n.s. † / ††	n.s. † / ††	n.s. † / ††	NA	n.s. † / ††	n.s. † / ††
**QoL**	n.s † / ††	n.s. † / ††	n.s † / ††	n.s. † / ††	n.s. † / ††	*8 weeks (shortest) higher E.S. 20 weeks (longest lowest E.S ††	n.s. † / ††	NA	**Low baseline, higher E.S. † / ††	n.s. † / ††	n.s. † / ††
**PG-YBOCS**	**Recent higher E.S. † / ††	*supplement higher E.S. † / ††	n.s † / ††	n.s. † / ††	n.s. † / ††	n.s. † / ††	NA	n.s. † / ††	**High baseline, higher E.S. † / ††	n.s. † / ††	n.s. † / ††
**GSAS**	.Recent Higher E.S. †	**ORAs higher E.S. *supplement higher E.S. ††	**No sponsor higher E.S. † / ††	n.s. † / ††	n.s. † / ††	n.s. † / ††	NA	NA	n.s. † / ††	n.s. †	n.s. † / ††
**CGI**	n.s † / ††	*AP and supplement lower E.S. ††	n.s † / ††	n.s. † / ††	n.s. † / ††	·6 weeks (shortest) lower E.S. ††	NA	n.s. † / ††	n.s. † / ††	n.s. †	n.s. † / ††

PG-YBOCS = Yale-Brown Obsessive-Compulsive Scale adapted for Pathological Gambling, GSAS = Gambling Symptom Assessment Scale, CGI-I = Clinical Global Impression-Improvement scale; QoL = Quality of life; E.S = effect size; n.s.= non-significant statistically; NA = not available/not appropriate; ORAs = Opiate receptor antagonists; MS = mood stabilisers; AP = antipsychotics; AD = antidepressants; COI = Conflict of Interest (defined as the presence of any declared industry-related conflict of interest by the authors in any section of the published manuscript); statistical significance: n.s. = non-significant; ‘.’ <0.10 (trend); ‘*’ <.05; ‘**’ <0.01; ‘***’ <0.001; NA = not available/not applicable; † = maintained in sensitivity analyses with *r* = 0.25; †† = maintained in sensitivity analyses with *r* = 0.75.

### Correlation between the placebo effects and the treatment effects

We calculated the correlation between the placebo effect sizes and the respective effect sizes for the treatment arms in each meta-analysis. For gambling severity the placebo and treatment effect sizes were highly correlated in all analyses (see Table 3); however, this was not true for quality of life meta-analyses, in which the placebo and treatment effect sizes were weakly correlated.

**Table 3.** Correlation between effect sizes (Pearson)

	Placebo gambling severity (overall)	Placebo QOL	Placebo PG-YBOCS	Placebo GSAS	Placebo CGI
**Treatment gambling severity (overall)**	*r* = 0.668 **				
**Treatment QOL**		*r* = 0.17 n.s.			
**Treatment PG-YBOCS**			*r* = 0.774 **		
**Treatment GSAS**				*r* = 0.60 *	
**Treatment CGI**					*r* = 0.93 ***

Statistical significance: n.s. = non-significant; ‘.’ <0.10 (trend); ‘*’ <.05; ‘**’ <0.01; ‘***’ <0.001.

### Sensitivity analyses

Full results at *r* = 0.25 and *r* = 0.75 are presented in the supplementary materials (paragraph §S6 & §S7). Sensitivity analyses did not substantially alter the results (see also Table 2 in which meta-regression analysis results maintained in sensitivity analyses are shown in tabularised format). The effect sizes were notably lower ‘overall’ at lower imputed correlations and higher overall at higher imputed correlation (i.e. SMCC for gambling severity was 1.00, 1.18 and 1.55 at *r* = 0.25, 0.50 and 0.75 respectively and SMCC for QOL was 0.51, 0.63 and 0.87 at *r* = 0.25, 0.50 and 0.75 respectively), however remained large in size for gambling severity and moderate-large for quality of life. Heterogeneity results were effectively similar in sensitivity analyses. Publication biases became more prominent at higher imputed correlations and the trim and fill method was required to produce more conservative (lower) effect estimates at *r* = 0.75 for most of the effect size estimates (see supplementary material §S7). The direction of correction from trim and fill was always towards the effects reported in the main paper (with *r* = 0.50).

### Nocebo effects

Due to paucity of data, those were calculated for each pharmacological class separately. Results indicated 2.4% drop outs from placebo arms in the antidepressant trials (fluvoxamine, paroxetine), 6.1% placebo drop outs for antipsychotics (olanzapine), 1.9% placebo drop outs for opiate receptor antagonists (naltrexone, nalmefene, naloxone), 1.6% placebo drop outs for mood stabilisers (topiramate, lithium) and 0% placebo drop outs for supplements (NAC, silymarin). Detailed results are presented in the supplement (paragraph §S8)

### Quality assessment

We completed RoB2 for all papers under scope. All domains had ‘low concern’ as the most common outcome, apart from Domain 5 (bias in selection of the reported result) in which ’some concern’ was the predominant outcome. Consequently, the majority of papers scored as ’some concern’ in the overall risk of bias. Full RoB2 scores are reported in the supplement (paragraph §S9).

## Discussion

This is the first systematic review and meta-analysis of placebo effects in RCTs for the pharmacological management of gambling disorder. Our findings indicate that placebo effects are prominent and of large magnitude for clinical efficacy outcomes of gambling severity (across scales), and of moderate-large magnitude for quality of life clinical efficacy measures. We first consider the implications of these high placebo effects, and then discuss moderating factors, and nocebo effects.

Placebo response rates can be influenced by the condition being studied. For example, placebo response rates in clinical trials are generally relatively high for depression, panic disorder, or generalised anxiety disorder but are typically low for certain other mental health conditions such as OCD or schizophrenia (e.g. see (Bernstein, Brown, Professor of Psychiatry, & Behavior, 2017; Cao *et al*., 2021; Jones *et al*., 2021; Huneke *et al*., 2024)). Our finding of a relatively high placebo response rate for gambling disorder bears similarity with what has been found for other addictions such as alcohol use disorder, where high placebo response rates have been noted (Anton *et al*., 2006; Del Re, *et al.,* 2013; Scherrer *et al*., 2021), and places gambling disorder among the mental disorders with the largest placebo effect sizes (Huneke *et al*., 2024). The finding of a high placebo response rate for gambling disorder has a number of implications. Firstly, the results suggest that people who seek treatment for gambling disorder experience notable symptom improvement that is not directly related to the active medication compound being examined – from the perspective of patient outcomes this is a positive, and highlights the potential importance of factors such as therapeutic alliance and non-specific support from research and clinical teams. Secondly, large placebo response potentially represents a design problem for pharmacological clinical trials of gambling disorder, by making it challenging to detect a true active effect of medication. Intriguingly, we found that quality of life measures show a greater disparity between placebo and treatment response. How different outcome measures affect estimates of treatment effect size needs to be further understood. The high placebo response also highlights a disparity in the literature for how different gambling disorder treatment modalities are compared (or contrasted) with each other. In particular, many psychological intervention clinical trials in gambling disorder have used waiting list control (Petry, *et al*., 2017). In practical terms, this suggests that pharmacological options should be considered more often in clinical practice, as they have had to pass a higher clinical standard to show their efficacy in trials. It is likely that randomisation to wait list results in reduced symptom improvement compared with a placebo due to fewer clinical interactions and lower expectations of benefit and/or ‘disappointment’ (Bandelow *et al*., 2015), thereby substantially inflating effect sizes for active therapy versus control. This issue of inflating treatment effect sizes by using a weak control condition has also been noted for other compulsive conditions such as OCD (Laws, *et al.,* 2022). In contrast, controlled clinical trials of medications have used placebo control, which is much more conservative and scientifically rigorous. This issue needs to be considered when weighing the balance in favour of particular treatment modalities for gambling disorder. Another consideration is around risks – with both medications and therapies being associated with adverse effects for some individuals, yet adverse events are almost always documented in pharmacological trials but rarely in psychological trials in the field (Klatte, *et al.*, 2023).

Little is yet known about the neurobiological determinants of the placebo effects in pharmacological trials of gambling disorder. We know that, in general, the placebo effect is mediated by diverse neurobiological processes, including learning, expectation and social cognition (Wager & Atlas, 2015). Commonalities in the neurobiological characteristics of gamblers, for example heightened impulsivity (Ioannidis, *et al.,* 2019) or a dysregulated anticipatory dopaminergic response (Linnet, 2020) could predispose to heightened placebo or nocebo responses, by altering underlying learning and reward expectation processes. Future studies could further investigate those neurobiological processes involved in the production of placebo and nocebo effects in gambling disorder.

### Study design elements, including blinding integrity

Study duration significantly moderated placebo response specifically for one symptom severity measure (CGI) and also for quality of life: with shorter duration linked to lower placebo effect on CGI, and linked to larger placebo effect on quality of life. The reasons for these results are unclear. We did not find that unbalanced randomisation moderated any of the meta-analyses effects.

In addition, placebo run-in (present in 38.9% or 7 out of 18 studies) did not significantly moderate the placebo response, indicating that inclusion of such a run-in did not help minimise placebo effect size – contrary to what might be anticipated. Nonetheless, this accords with findings in antidepressant trials, where placebo run-in periods are associated with reductions in treatment response in *both* arms and thereby not altering the efficacy comparison between medication and placebo (Scott, *et al*., 2022). Given that using placebo run-in periods does not minimise placebo effect size, could affect the external validity of the trial, and necessarily involves deception which could be considered unethical, we would recommend against their use in pharmacological trials for gambling disorder.

We also found that none of the studies assessed blinding success (i.e. through debriefing of participants and/or research staff). One study made a specific effort to prevent side effects (J. E. Grant, *et al*., 2008) and utilised an independent investigator at expected peak of side effects to avoid un-blinding due to common side effects.

### Medication class

Turning now to significant moderators of placebo effects in this study, we found that the supplements medication class, here including n-acetyl cysteine (NAC) and silymarin, moderated a higher placebo effect size for symptom severity measures overall. This is interesting in that supplement medications are often perceived by service users as ‘innocuous’ or not likely to have any side effects (Ernst, 1998). Stronger placebo responses might occur because individuals perceive supplements as a ‘more natural’ way to correct symptoms or more acceptable. Another possibility is that due to relatively good side effect profiles observed for these supplements in clinical trial conditions, blinding may have been ‘truer’ as compared to studies of other classes of medications that are more likely to have side effects. However, we currently do not have the data to make inferences regarding the effect that side effects or beliefs might have on blinding and thus estimates of efficacy. Future studies could address the success or otherwise of blinding by debriefing both study participants and investigators after trial completion with standard instruments (Haq, *et al*., 2024).

### Recency of publication

We found that later year of publication predicted higher placebo effect sizes, for symptom severity measures overall. One could expect that newer studies would follow more rigorous approaches to standardising placebo procedures, leading to diminished or stable placebo effects, however this is not the case here. Interestingly, across psychiatric disorders in general, it has been shown that placebo effects have been larger with more recent publications (Weimer *et al*., 2015; Huneke *et al*., 2024). The reasons for this change are simply not known, but findings herein for gambling disorder are consistent with those in many other mental health conditions.

### Industry influence

We found that the absence of company sponsorship was associated with higher effect sizes in the placebo arms for symptom severity measures overall. This is a novel result with a lot of interesting implications. It is possible that participants being aware of company sponsorship are negatively influenced towards the beneficial effects of the medication treatment, thus generating a lesser placebo response. However, it has also been shown that industry sponsorship is associated with higher effect sizes in RCTs in general (Lundh, *et al.,* 2017), in which case this applies to both treatment and placebo arms, which are highly correlated in this dataset. Another possibility is that clinical trials conducted in academic settings may offer more additional support (irrespective of randomisation) as contrasted to commercial studies potentially involving generic / non-specialist recruitment sites, such as spending time with expert clinicians who take time to speak with patients or offer formal psychological support. Interestingly, self-reported industry-related conflict of interest from the manuscripts authors did not moderate any effect sizes, meaning that we did not find any evidence that those declared relationships from the authors were associated with the effect sizes reported. It is possible that moderation by absence of industry sponsorship was related to the moderation by medication class, as both studies which included supplements were unsponsored.

### Choice of severity instrument, baseline severity

While placebo effects were numerically larger in the studies which assessed gambling severity improvements via clinician structured instruments, than self-reported instruments, or unstructured clinical instruments, choice of severity instrument was not statistically significant in moderation analyses, suggesting that approaches similarly capture placebo effects, in terms of gambling severity. However, those scales had differences as well; for example, we found higher baseline symptom severity leading to higher placebo response, using the PG-YBOCS instrument, which was also true for the treatment arms, using both PG-YBOCS and GSAS. That is an interesting finding, which may suggest that some placebo effects follow a ‘regression to the mean’ pattern (Cummings *et al*., 2004), that is can be attributed to statistical artefacts which are unequally distributed across levels of baseline severity, or that they are impacted by floor effects. Furthermore, it is interesting that the reverse has been shown prior to the current study, for placebo effects in alcohol dependence RCTs (Scherrer *et al*., 2021) and quality of life followed the same reverse pattern (lower baseline was associated with higher effect seizes) so this result merits exploration in future work.

### Nocebo effects

In terms of nocebo effects, low placebo dropout rates (<2%) were found for clinical trials focusing on supplements (NAC, silymarin), mood stabilisers, and opioid receptor antagonists; slightly higher for SSRIs (2.4%); and higher rates were observed for placebo drop outs for antipsychotic (olanzapine) studies (6.1%). This variability could reflect expectation (e.g. people may anticipate ‘worse’ side effects for olanzapine had they read about it in advance, not knowing if they were then assigned to active or placebo treatment (Faasse *et al*., 2019)). Low nocebo effects is useful because it reduces clinical trial drop out, which can undermine the integrity of clinical trials.

### Limitations

Several possible limitations should be considered, reflecting both limitations of the included RCTs and of our meta-analysis. In terms of the included RCTs, the number available for gambling disorder is relatively limited to date, as compared to other areas of mental ill health (e.g. depression). The reasons for this are multi-fold, and include a historical (and persisting) lack of funding for gambling disorder research from independent national funding schemes globally. Available RCTs also have a number of methodological issues such as relatively small sample sizes (in many cases) as compared to other areas of mental ill health. The available studies did not generally examine views towards medications (e.g. pharmaceuticals versus nutraceuticals), expectation, or other variables (e.g. personality) that may relate to placebo response. We also identified a moderate-high degree of heterogeneity, particularly in the meta-analyses of gambling severity using the PG-YBOCS scale; this might reflect differences in other elements of study design (e.g. duration, presence of comorbidities etc). Specifically, when it comes to the placebo effect, the presence of comorbidities may play a role. Particularly axis-II issues may influence placebo responses (Yadav, 2020) and those have been under-investigated in gambling disorder RCTs.

In terms of limitations of the meta-analysis, we could not consider time course of placebo responses – for example, we could not establish who responded to placebo for the whole of a given study as opposed to responding just at the end. The meta-regression analyses could not be conducted for all *a priori* hypothesised predictors due to lack of data in given categories (e.g. no study assessed blinding integrity); and it should also be considered that the number of studies in the explored moderator categories was small in some cases (e.g. having only one RCT with unbalanced randomisation), potentially limiting power to detect effects of moderators. The other limitation of the moderation analyses is that those were not corrected for multiple comparisons, which suggests that those should be considered as exploratory, requiring further investigation once (if) a substantially larger number of clinical trials become available for analysis in the future. Finally, one of the limitations of examining nocebo effects in this analysis is that we were only able to explore the aspect of nocebo effect which led to drop out from the trials. Other, potentially more minor nocebo effects, which may though influence clinical efficacy or blinding success, were not consistently reported to allow for robust examination. Future studies should maintain a consistent study design and reporting, to allow for the reduction of heterogeneity, but also to include study design elements like the assessment of comorbidities, assessment of blinding success and nocebo effect details, to aid the interpretation of future findings.

## Conclusions and implications for future research

The current meta-analysis provides insights into the importance of placebo effects in pharmacological RCTs for gambling disorder. Considering these effects is also of relevance to routine clinical practice, for example, being aware of placebo effects may encourage clinicians to spend more time addressing a patient’s beliefs and attitudes around medication before starting treatment to enhance chances they positively respond. We found that placebo effects are prominent (large) across these trials and that there are several moderators, on at least some outcome measures of these placebo effects (year of publication, medication class, duration of treatment, company sponsorship status). Nocebo effects were measurable and may be influenced by medication class being studied in a particular trial. The study also found a lack of assessment of blinding integrity across the available RCTs and unclear use of independent raters. Future studies should consider those parameters when designing RCTs for the assessment of clinical efficacy for the pharmacological management of gambling disorder. It remains unclear how best to minimise (or at least make predictable) placebo effects in clinical trials for gambling disorder, because placebo ‘run-in’ did not significantly moderate placebo response herein. Finally, we recommend that future RCTs of psychological interventions for gambling disorder adopt rigorous control conditions and measure adverse events – to ensure parity with the standards already being set by pharmacological RCTs in the field.

## Supporting information

Ioannidis et al. supplementary material Ioannidis et al. supplementary material

## Data Availability

The data that support the findings of this study are available upon reasonable request from the corresponding author (e.g. for peer review purposes) and will be published upon acceptance of the manuscript.

## Supplementary material

The supplementary material for this article can be found at https://doi.org/10.1017/neu.2024.52.

## Analytic code availability

Example R code and package information for meta-analysis can be found in the supplementary material (§S10).
