# Supplementary material for: Placebo and nocebo effects in gambling disorder pharmacological trials: a meta-analysis
Source: Acta Neuropsychiatr. 2024 Nov 20;37:e40. doi: 10.1017/neu.2024.52 (PMC13130306; doi:10.1017/neu.2024.52)
Supplement: Ioannidis et al. supplementary material [file S0924270824000528sup001.docx]

## §S1 Forest plots for treatment arms


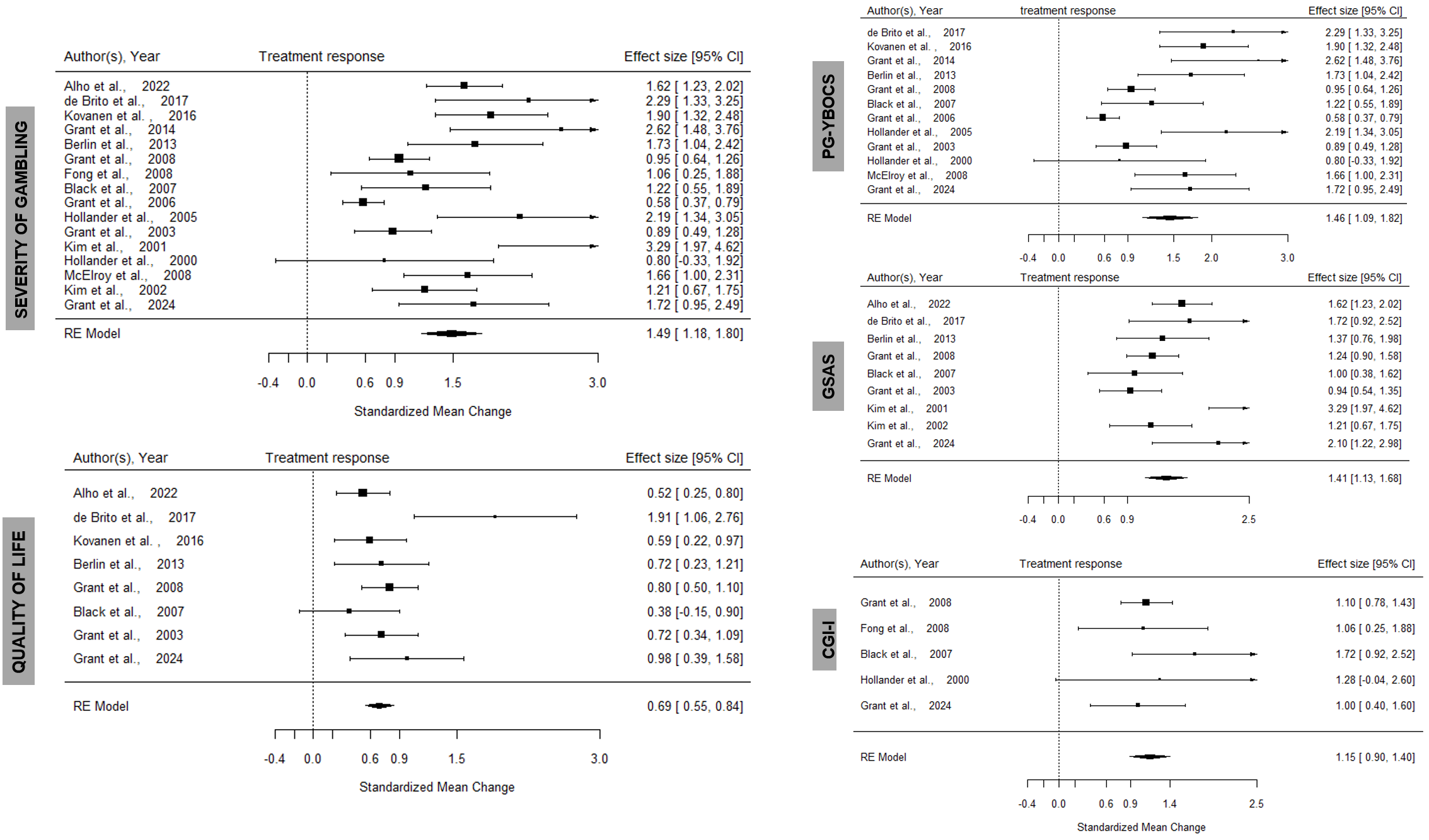


## §S2 Heterogeneity measures for treatment arms

|  | **tau^2 (estimated amount of total heterogeneity):** | **tau (square root of estimated tau^2 value):** | **I^2 (total heterogeneity / total variability):** | **H^2 (total variability / sampling variability):** | **Test for Heterogeneity:** |
| --- | --- | --- | --- | --- | --- |
| **Treatment gambling severity** | 0.2709 (SE = 0.1421) | 0.5205 | 79.37% | 4.85 | Q(df = 15) = 78.1221, p-val < .0001 |
| **Treatment QOL** | 0.0028 (SE = 0.0221) | 0.0534 | 6.14% | 1.07 | Q(df = 7) = 12.3856, p-val = 0.0886 |
| **Treatment PGYBOCS** | 0.2859 (SE = 0.1718) | 0.5347 | 80.77% | 5.20 | Q(df = 11) = 59.1895, p-val < .0001 |
| **Treatment GSAS** | 0.0839 (SE = 0.0837) | 0.2896 | 52.83% | 2.12 | Q(df = 8) = 19.4638, p-val = 0.0126 |
| **Treatment CGI** | 0  (SE = 0.0638) | 0 | 0.00% | 1.00 | Q(df = 4) = 2.3691, p-val = 0.6682 |

**Legend**: PG-YBOCS = Yale-Brown Obsessive Compulsive Scale adapted for Pathological Gambling, GSAS = Gambling Symptom Assessment Scale, CGI-I = Clinical Global Impression-Improvement scale; SE = standard error; df = degrees of freedom

## §S3 Publication bias in the treatment arms


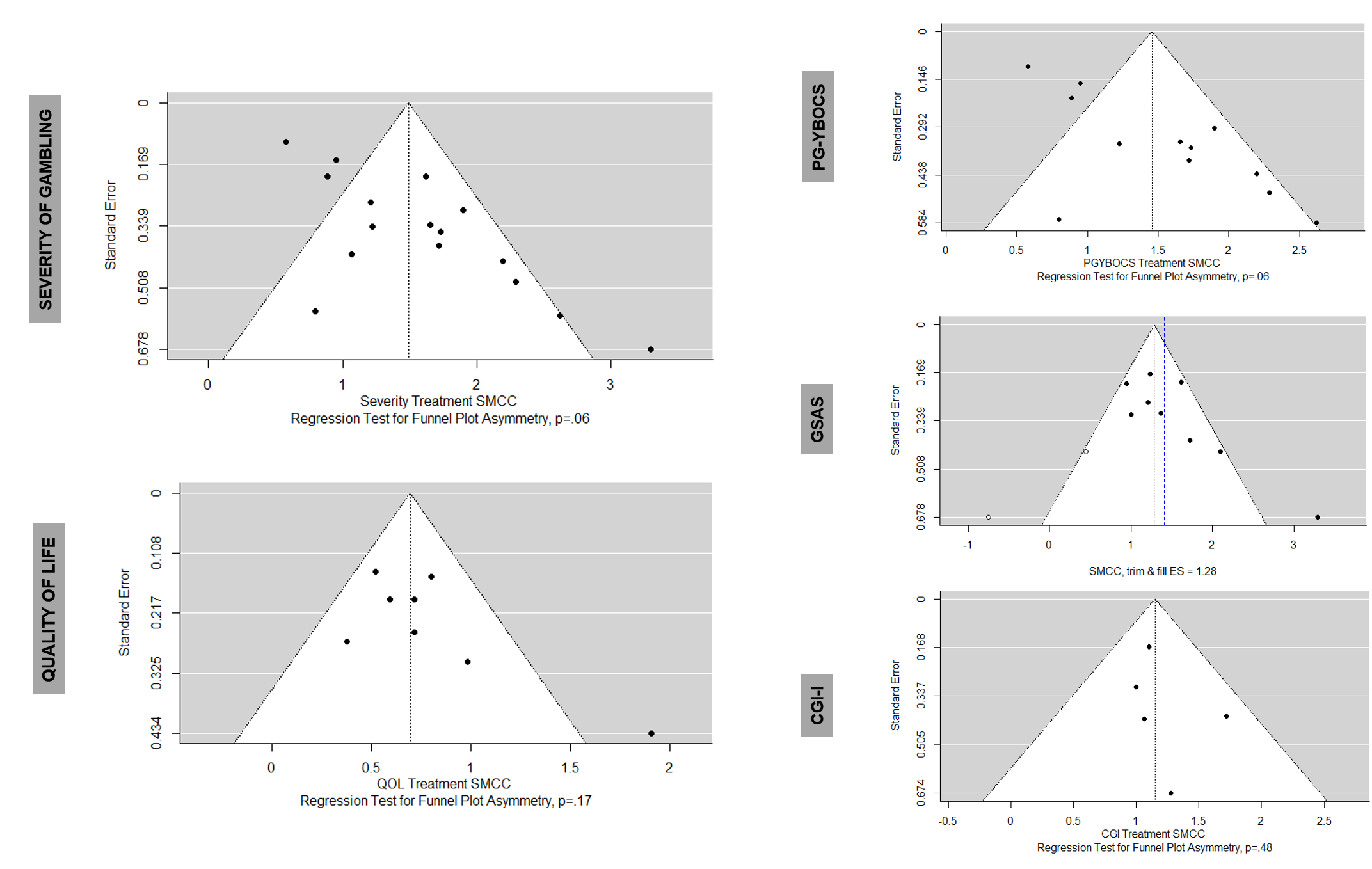


**Legend** – Funnel plots with regression for funnel plot asymmetry p-values. PG-YBOCS = Yale-Brown Obsessive Compulsive Scale adapted for Pathological Gambling, GSAS = Gambling Symptom Assessment Scale, CGI-I = Clinical Global Impression-Improvement scale. Publication bias identified in the Quality of life analysis and the trim and fill method was used to provide a new effect size estimate.

## §S4 Meta-regression analyses (meta-regression) – TREATMENT effects

| **Analysis** | **Publication Year** | **Medication type (class)** | **Sponsored study (Y/N)** | **Participant age (mean)** | **%gender** | **Duration of study** | **Unbalanced randomization** | **Baseline severity** | **Severity scale** | **Author COI (Y/N)** | **Placebo run in 1 week** |
| --- | --- | --- | --- | --- | --- | --- | --- | --- | --- | --- | --- |
| **Severity (all measures)** | n.s. | * MS higher E.S.  ·  supplement higher E.S. | n.s. | n.s. | n.s. | ** 16-weeks, lower E.S. * 17-weeks lower E.S. | n.s. | NA | n.s. | n.s. | n.s. |
| **PGYBOCS** | *  Recent higher E.S. | * MS higher E.S.  * supplement higher E.S. | n.s | n.s. | n.s. | ** 16-weeks, lower E.S. * 17-weeks, lower E.S. | n.s. | *** higher baseline higher E.S. | NA | n.s. | n.s. |
| **GSAS** | *  Recent higher E.S. | * supplement higher E.S. | n.s. | n.s. | n.s. | ** 11-week (the shortest) duration higher E.S. | NA | * higher baseline higher E.S. | NA | n.s. | n.s. |
| **CGI** | n.s | n.s. | n.s | n.s. | n.s. | n.s. | n.s. | n.s. | NA | n.s. | n.s. |
| **QoL** | n.s | n.s. | n.s | * higher age higher E.S. | n.s. | n.s. | NA | NA | n.s. | *  Presence of COI lower E.S. | n.s. |

**Legend**: PGYBOCS=Problematic Gambling Yale-Brown Obsessive Compulsive Scale (clinician rated, structured); GSAS = Gambling Symptom Assessment Scale (self-reported); CGI = Clinical Global Improvement (clinician scored – unstructured); QoL = Quality of life; E.S = effect size; n.s.= non-significant statistically; ORAs = Opiate receptor antagonists; MS = mood stabilizers; AP = antipsychotics; AD = antidepressants; statistical significance: n.s. = non-significant; ‘.’ <.10 (trend); ‘*’ <.05; ‘**’ <0.01; ‘***’ <0.001; NA = not available/not applicable

## §S5 Correlation between effect sizes (Pearson)

|  | **Placebo gambling severity** | **Placebo QOL** | **Placebo PGYBOCS** | **Placebo GSAS** | **Placebo CGI** |
| --- | --- | --- | --- | --- | --- |
| **Treatment gambling severity** | r=0.669  ** |  |  |  |  |
| **Treatment QOL** |  | r=0.198  n.s. |  |  |  |
| **Treatment PGYBOCS** |  |  | r=0.735  ** |  |  |
| **Treatment GSAS** |  |  |  | r=0.672  * |  |
| **Treatment CGI** |  |  |  |  | r=0.96  *** |

## §S6 Sensitivity analysis with correlation 0.25

### Forest plots


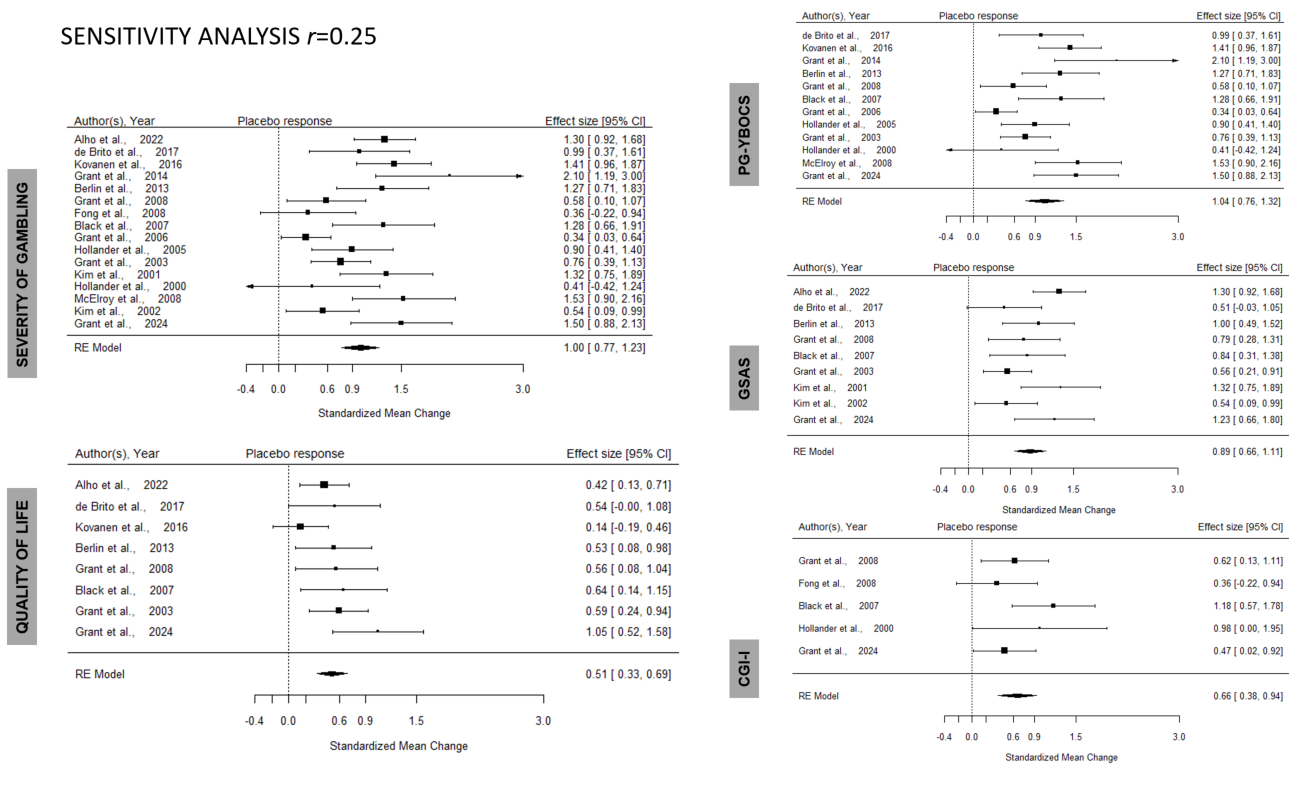


### Funnel plots


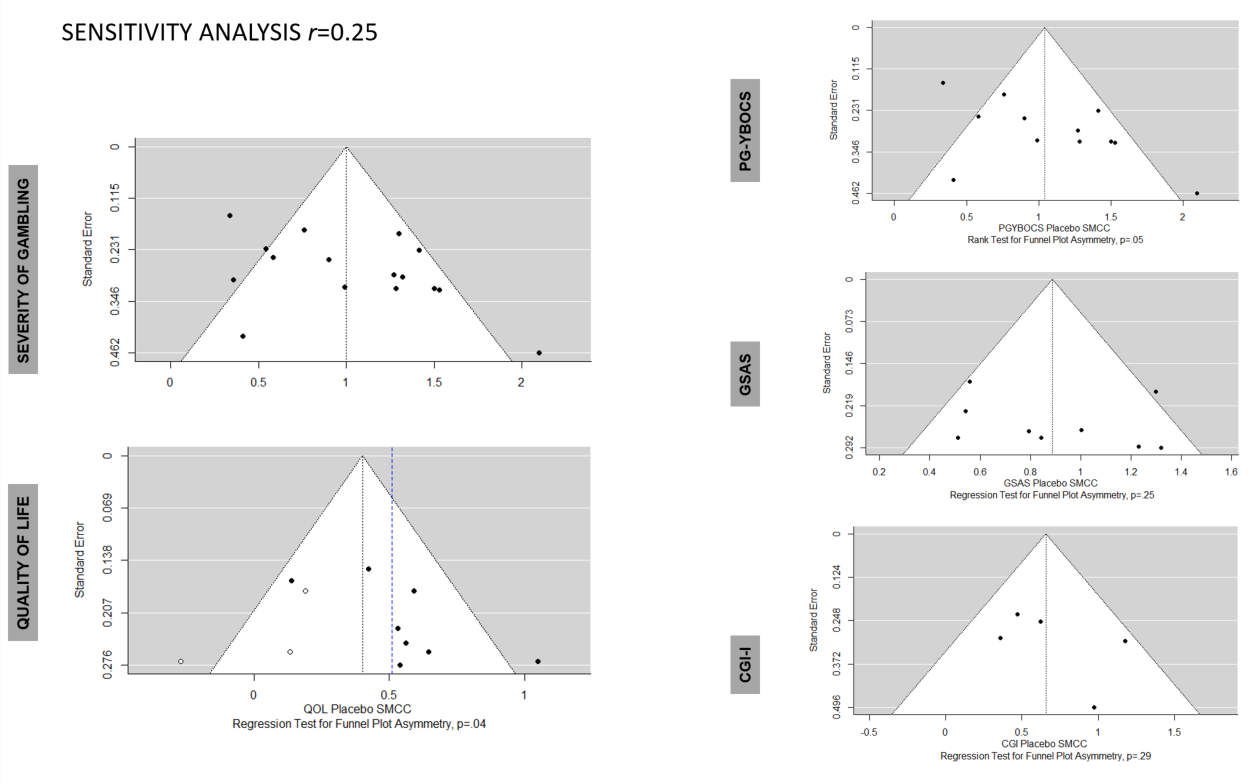


**Legend** – Funnel plots with regression for funnel plot asymmetry p-values. PG-YBOCS = Yale-Brown Obsessive Compulsive Scale adapted for Pathological Gambling, GSAS = Gambling Symptom Assessment Scale, CGI-I = Clinical Global Impression-Improvement scale. Publication bias identified in the Quality of life analysis and the trim and fill method was used to provide a new effect size estimate.

### Heterogeneity placebo with r=0.25

|  | **tau^2 (estimated amount of total heterogeneity):** | **tau (square root of estimated tau^2 value):** | **I^2 (total heterogeneity / total variability):** | **H^2 (total variability / sampling variability):** | **Test for Heterogeneity:** |
| --- | --- | --- | --- | --- | --- |
| **Placebo gambling severity** | 0.1464 (SE = 0.0808) | 0.3826 | 69.19% | 3.25 | Q(df = 15) = 51.0825, p-val < .0001 |
| **Placebo QOL** | 0.0191 (SE = 0.0333) | 0.1383 | 30.57% | 1.44 | Q(df = 7) = 9.7669, p-val = 0.2022 |
| **Placebo PGYBOCS** | 0.1565 (SE = 0.1010) | 0.3956 | 69.57% | 3.29 | Q(df = 11) = 39.0654, p-val < .0001 |
| **Placebo GSAS** | 0.0572 (SE = 0.0586) | 0.2391 | 49.47% | 1.98 | Q(df = 8) = 16.0486, p-val = 0.0417 |
| **Placebo CGI-I** | 0.0183 (SE = 0.0713) | 0.1352 | 17.54% | 1.21 | Q(df = 4) = 4.9326, p-val = 0.2943 |

**Legend**: PG-YBOCS = Yale-Brown Obsessive Compulsive Scale adapted for Pathological Gambling, GSAS = Gambling Symptom Assessment Scale, CGI-I = Clinical Global Impression-Improvement scale; SE = standard error; df = degrees of freedom

### Meta-regression, placebo with r=0.25

| **Analysis** | **Publication Year** | **Medication type (class)** | **Sponsored study (Y/N)** | **Participant age (mean)** | **%gender** | **Duration of study** | **Unbalanced randomization** | **Baseline severity** | **Severity scale** | **Author COI (Y/N)** | **Placebo run in 1 week** |
| --- | --- | --- | --- | --- | --- | --- | --- | --- | --- | --- | --- |
| **Severity (all measures)** | *  Recent higher E.S. | *  supplement higher E.S. | n.s. | n.s. | n.s. | n.s. | n.s. | NA | n.s. | n.s. | n.s. |
| **QoL** | n.s | n.s. | n.s | n.s | n.s. | n.s. | NA | ** lower baseline higher E.S. | n.s. | n.s. | n.s. |
| **PGYBOCS** | *  Recent higher E.S. | * supplement higher E.S. | n.s | n.s. | n.s. | n.s. | n.s. | ** higher baseline higher E.S. | NA | n.s. | n.s. |
| **GSAS** | n.s. | **  ORA higher E.S. | ** no sponsor higher E.S. | n.s. | n.s. | n.s. | NA | n.s. | NA | n.s. | n.s. |
| **CGI** | n.s | n.s. | n.s | n.s. | n.s. | n.s. | n.s. | n.s. | NA | n.s. | n.s. |

**Legend**: PG-YBOCS = Yale-Brown Obsessive Compulsive Scale adapted for Pathological Gambling, GSAS = Gambling Symptom Assessment Scale, CGI-I = Clinical Global Impression-Improvement scale; QoL = Quality of life; E.S = effect size; n.s.= non-significant statistically; NA = not available/not appropriate; ORAs = Opiate receptor antagonists; MS = mood stabilizers; AP = antipsychotics; AD = antidepressants; COI = Conflict of Interest (defined as the presence of any declared industry related conflict of interest by the authors in any section of the published manuscript); statistical significance: n.s. = non-significant; ‘.’ <.10 (trend); ‘*’ <.05; ‘**’ <0.01; ‘***’ <0.001; NA = not available/not applicable;

## §S7 Sensitivity analysis with correlation 0.75

### Forest plots


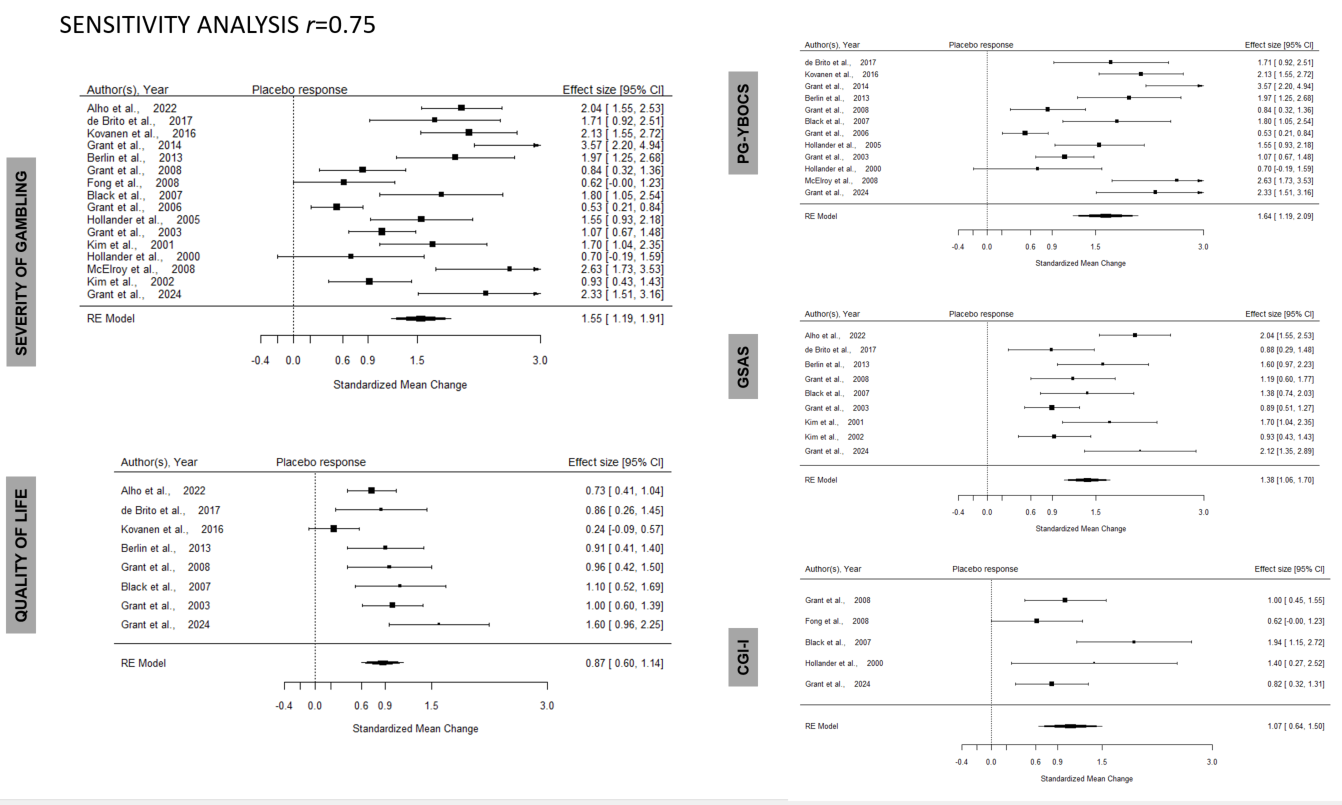


### Funnel plots


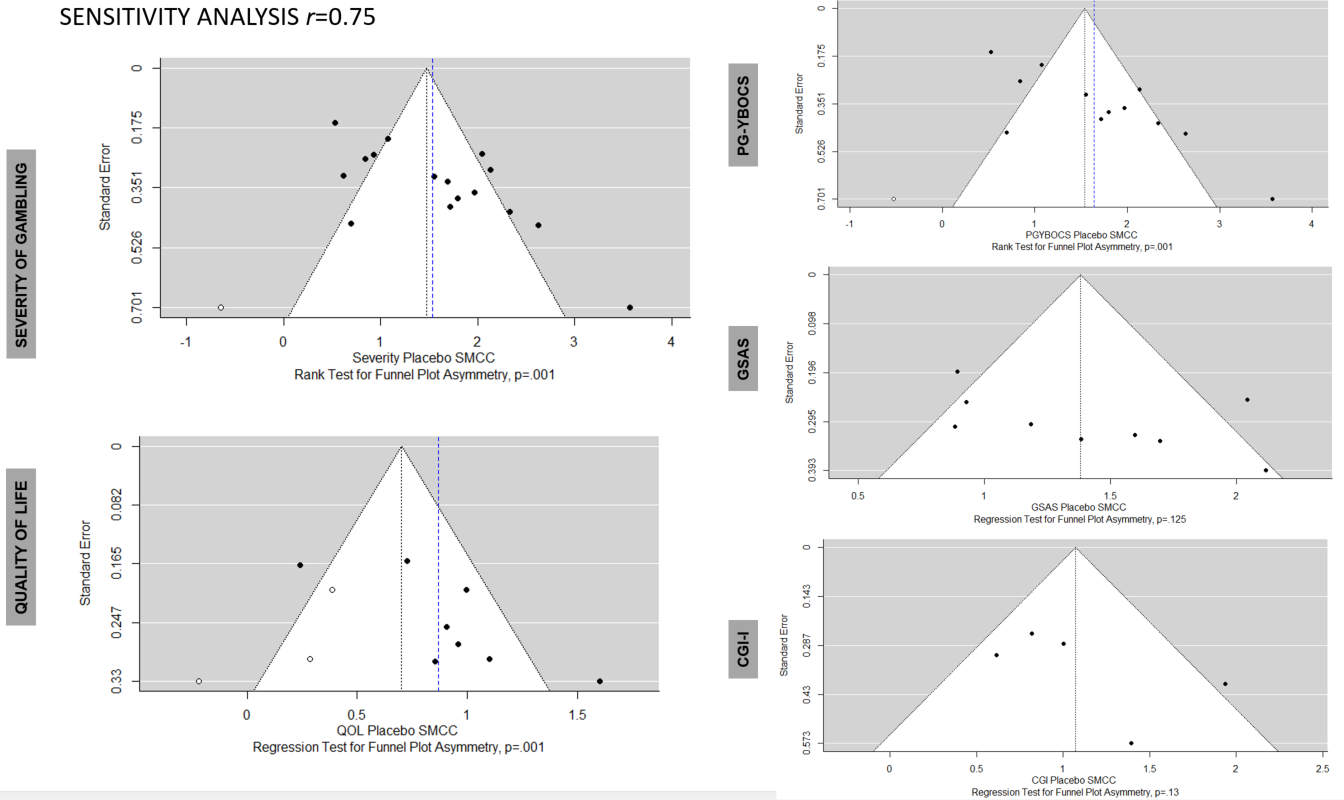


**Legend** – Funnel plots with regression for funnel plot asymmetry p-values. PG-YBOCS = Yale-Brown Obsessive Compulsive Scale adapted for Pathological Gambling, GSAS = Gambling Symptom Assessment Scale, CGI-I = Clinical Global Impression-Improvement scale. Publication bias identified in the Quality of life analysis and the trim and fill method was used to provide a new effect size estimate.

### Heterogeneity placebo with r=0.75

|  | **tau^2 (estimated amount of total heterogeneity):** | **tau (square root of estimated tau^2 value):** | **I^2 (total heterogeneity / total variability):** | **H^2 (total variability / sampling variability):** | **Test for Heterogeneity:** |
| --- | --- | --- | --- | --- | --- |
| **Placebo gambling severity** | 0.4269 (SE = 0.1989) | 0.6534 | 82.64% | 5.76 | Q(df = 15) = 85.3534, p-val < .0001 |
| **Placebo QOL** | 0.0908 (SE = 0.0797) | 0.3014 | 63.37% | 2.73 | Q(df = 7) = 19.8945, p-val = 0.0058 |
| **Placebo PGYBOCS** | 0.4976 (SE = 0.2690) | 0.7054 | 83.89% | 6.21 | Q(df = 11) = 68.1753, p-val < .0001 |
| **Placebo GSAS** | 0.1507 (SE = 0.1185) | 0.3882 | 65.07% | 2.86 | Q(df = 8) = 23.8891, p-val = 0.0024 |
| **Placebo CGI-I** | 0.1215 (SE = 0.1686) | 0.3486 | 52.30% | 2.10 | Q(df = 4) = 7.9725, p-val = 0.0926 |

**Legend**: PG-YBOCS = Yale-Brown Obsessive Compulsive Scale adapted for Pathological Gambling, GSAS = Gambling Symptom Assessment Scale, CGI-I = Clinical Global Impression-Improvement scale; SE = standard error; df = degrees of freedom

### Meta-regression, placebo with r=0.75

| **Analysis** | **Publication Year** | **Medication type (class)** | **Sponsored study (Y/N)** | **Participant age (mean)** | **%gender** | **Duration of study** | **Unbalanced randomization** | **Baseline severity** | **Severity scale** | **Author COI (Y/N)** | **Placebo run in 1 week** |
| --- | --- | --- | --- | --- | --- | --- | --- | --- | --- | --- | --- |
| **Severity (all measures)** | **  Recent higher E.S. | *  supplement higher E.S. | n.s. | n.s. | n.s. | n.s. | n.s. | NA | n.s. | n.s. | n.s. |
| **QoL** | n.s | n.s. | n.s | n.s | n.s. | * 8 weeks higher E.S.  ** 20-weeks lower E.S. | NA | *** lower baseline higher E.S. | n.s. | n.s. | n.s. |
| **PGYBOCS** | **  Recent higher E.S. | * supplement higher E.S. | n.s | n.s. | n.s. | n.s. | n.s. | *  higher baseline higher E.S. | NA | n.s. | n.s. |
| **GSAS** | *  Recent higher E.S. | *  ORA higher E.S.  * supplement higher E.S. | **  no sponsor higher E.S. | n.s. | n.s. | n.s. | NA | n.s. | NA | * absence of COI higher E.S. | n.s. |
| **CGI** | n.s | *  AP lower E.S.  * supplement lower E.S. | n.s | n.s. | n.s. | ** 6-weeks, *8-weeks lower E.S. | n.s. | n.s. | NA | * absence of COI higher E.S. | n.s. |

**Legend**: PG-YBOCS = Yale-Brown Obsessive Compulsive Scale adapted for Pathological Gambling, GSAS = Gambling Symptom Assessment Scale, CGI-I = Clinical Global Impression-Improvement scale; QoL = Quality of life; E.S = effect size; n.s.= non-significant statistically; NA = not available/not appropriate; ORAs = Opiate receptor antagonists; MS = mood stabilizers; AP = antipsychotics; AD = antidepressants; COI = Conflict of Interest (defined as the presence of any declared industry related conflict of interest by the authors in any section of the published manuscript); statistical significance: n.s. = non-significant; ‘.’ <.10 (trend); ‘*’ <.05; ‘**’ <0.01; ‘***’ <0.001; NA = not available/not applicable;

## §S8 Nocebo effects

|  | type | n | drop outs from ‘medication’ attributed side effects | % nocebo for drop out outcomes |
| --- | --- | --- | --- | --- |
| 1 | AD | 83 | 2 | 0.02409639 |
| 2 | AP | 33 | 2 | 0.06060606 |
| 3 | MS | 59 | 1 | 0.01694915 |
| 4 | ORA | 152 | 3 | 0.01973684 |
| 5 | Supplement | 21 | 0 | 0.00000000 |

## §S9 Quality assessment (Risk of bias)


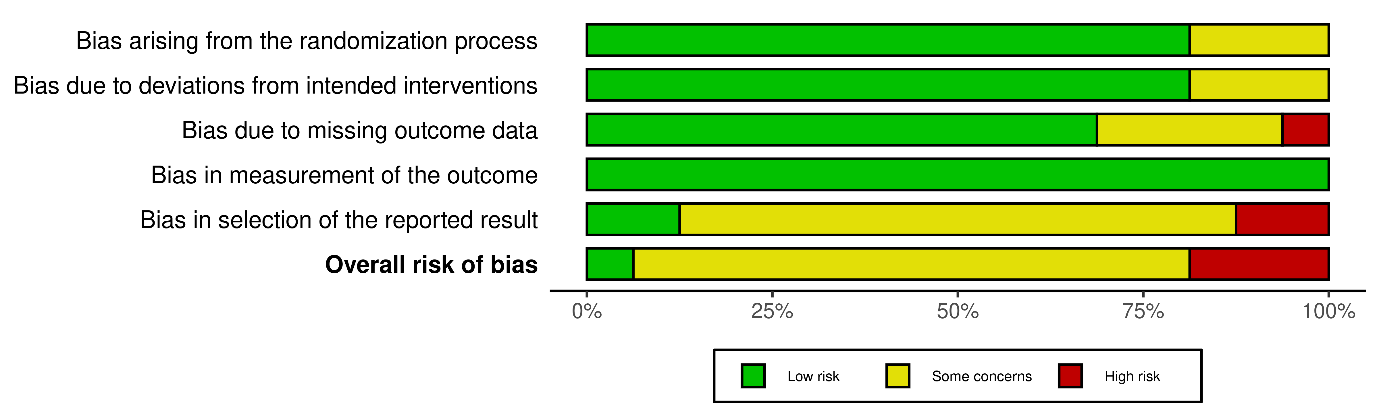


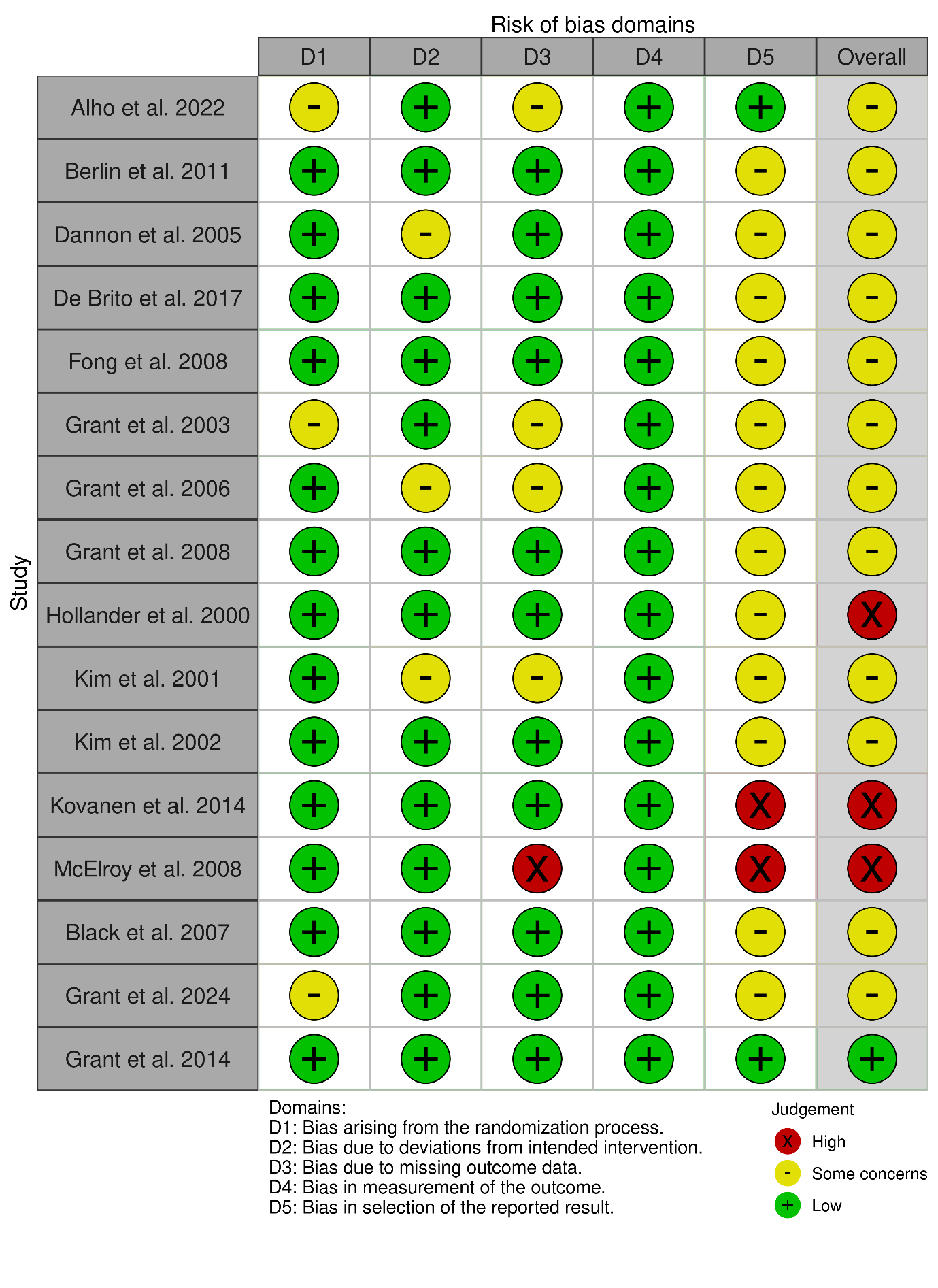


## §S10 R code

R version 4.2.1 (2022-06-23 ucrt) Platform: x86_64-w64-mingw32/x64 (64-bit) Running under: Windows 10 x64 (build 22631)

library(data.table)

library(metafor)

library(readxl)

library(meta)

library(robumeta)

library(dplyr)

df<- read.csv("~/Dataset.csv", head = TRUE, stringsAsFactors = FALSE)

df <- as.data.table(df); df <- df %>% filter(to.be.included == "1")

#placebo response of mixed severity of gambling results; follow similar for other domains/analyses.

placebo_meta <- df %>% filter(arm=="Placebo") %>% filter(symptom_sd_t0 != 0) %>%

dplyr::select(study_id, RCT_design_1, Publication.Year, type, sponsor, age_mean, perc_male, duration, scale, authors_COI, run_in, first_author, n_itt, symptom_mean_t0:sd_change..t1.t0.)

dat <- escalc(measure="SMCC", m1i=symptom_mean_t0, sd1i=symptom_sd_t0, m2i=symptom_mean_t1, sd2i=symptom_sd_t1, ni=n, ri=c(rep(0.50, 16)), data=placebo_meta, slab=paste(first_author, Publication.Year, sep=", ")) #impute correlation for sensitivity analyses

res <- rma(yi, vi, data=dat) ; predict(res, digits=3); confint(res)

forest(res, xlim=c(-2.6,4.6),

at=(c(-.4,-.2,0,.2,.6,.9, 1.5, 3)), digits=c(2,1), cex=.8)

text(-2.6, 17.5, "Author(s), Year", pos=4, cex=.9)

text(-0.6, 17.5, "Placebo response", pos=4, cex=.9)

text( 4.6, 17.5, "Effect size [95% CI]", pos=2, cex=.9)

plb1.yi <- res$yi; plb1.slab <- res$slab; b_res <- rma(yi, vi,data=dat, slab=study_id) #save results for further comparisons

#baujat(b_res); inf <- influence(res); print(inf); plot(inf) # influence diagnostics

### funnel plot

funnel(res, xlab = "Severity Placebo SMCC \nRank Test for Funnel Plot Asymmetry, p=XXX") #Tests for bias

regtest(res); ranktest(res); #for trim and fill if necessary

res.tf <- trimfill(res); res.tf

funnel(res.tf, xlab = "Severity Placebo SMCC \nRank Test for Funnel Plot Asymmetry, p=.XXX")

abline(v=1.54, col="blue", lwd=1, lty = 2)

#Meta-regression analysis for publication year, type, sponsor, age_mean, perc_male, duration, scale, authors_COI

res.modage <- rma(yi, vi, mods = ~ Publication.Year, data=dat)

res.modage

## List of papers included in the meta-analysis

| **#** | **First Author** | **Year** | **Journal** | **Study Type and Design** | **Arms** | **N randomized** | **Age (mean) /arm** | **%Male /arm** | **Route/ dosing scheme** | **Min. dose†** | **Max. dose†** | **Duration** | **Sponsorship** |
| --- | --- | --- | --- | --- | --- | --- | --- | --- | --- | --- | --- | --- | --- |
| **1** | Alho et al.(Alho et al., 2022) | 2022 | Add Behav | Double Blind RCT Parallel design, 2 arms | naloxone vs. placebo | 62/64 | 44.0 /  45.2 | 72% / 67% | Intranasal QDS/PRN | 4mg | 16mg | 12-weeks | No |
| **2** | De Brito et al.(de Brito et al., 2017) | 2017 | J Gambl Studies | Double Blind RCT Parallel design, 2 arms | topiramate vs. placebo | 18/20 | 50.6 /  45.8 | 53% / 53% | Oral BD | 25mg | 300mg | 12-weeks | Yes |
| **3** | Kovanen et al.(Kovanen et al., 2016) | 2016 | Eur Addict Res | Double Blind RCT Parallel design, 2 arms | naltrexone vs. placebo | 50/51 | 47.4 /  44.5 | 66% / 71% | Oral OD/PRN | 50mg | 50mg | 20-weeks | Yes |
| **4** | McElroy et al.(McElroy, Nelson, Welge, Kaehler, & Keck, 2008) | 2008 | J Clin Psychiatry | Double Blind RCT Parallel design, 2 arms | olanzapine vs. placebo | 21/21 | 51.5 /  46.8 | 38% / 48% | Oral, flexible | 2.5mg | 15mg | 12-weeks | Yes |
| **5** | Berlin et al.(Berlin et al., 2013) | 2013 | WJBP | Double Blind RCT Parallel design, 2 arms | topiramate vs. placebo | 20/22 | 50.5 /  44.9 | 50% / 45% | Oral, flexible | 25mg | 300mg | 14-weeks | Yes |
| **6** | Grant et al.(Grant, Kim, Hollander, & Potenza, 2008) | 2008 | J Clin Psychiatry | Double Blind RCT Parallel design, 2 arms | naltrexone vs. placebo | 58/19 | 47.8 /  44.7 | 36% / 47% | Oral, titration | 25mg | 150mg | 17-weeks | No |
| **7** | Fong et al.(Fong, Kalechstein, Bernhard, Rosenthal, & Rugle, 2008) | 2008 | Pharm Bio Beh | Double Blind RCT Parallel design, 2 arms | olanzapine vs. placebo | 11/12 | 46.6  43.6 | 55% / 50% | Oral, titration | 2.5mg | 10mg | 6-weeks | Yes |
| **8** | Black et al. (Black et al., 2007) | 2007 | J Clin Pharmacol | Double Blind RCT Parallel design, 2 arms | bupropion vs. placebo | 18/21 | 42.8  43.5 | 61% / 81% | Oral, flexible | 150mg | 375mg | 12-weeks | No |
| **9** | Grant et al.(Grant et al., 2006) | 2006 | Am J Psychiatry | Double Blind RCT Parallel design, 4 arms | nalmefene vs. placebo | 156/51 | 47.0 /  45.3 /  44.9 /  45.7/  46.3 | 54% / 56% / 50% / 67% | Oral, OD | 25mg | 100mg | 16-weeks | Yes |
| **10** | Kim et al.(Kim, Grant, Adson, & Zaninelli, 2002) | 2002 | J Clin Psychiatry | Double Blind RCT Parallel design, 2 arms | paroxetine vs. placebo | 23/22 | 49.3 /  49.3 | 43% / 28% | Oral, flexible | 20mg | 60mg | 8-weeks | Yes |
| **11** | Grant et al. (Grant et al., 2003) | 2003 | Int Clin Psychopharmacol | Double Blind RCT Parallel design, 2 arms | paroxetine vs. placebo | 36/40 | 47.0 /  42.0 | 44% / 75% | Oral, flexible | 10mg | 60mg | 16-weeks | Yes |
| **12** | Kim et al.(Kim, Grant, Adson, & Shin, 2001) | 2001 | Biol Psychiatry | Double Blind RCT Parallel design, 2 arms | naltrexone vs. placebo | 20/25 | 48.0 /  49.0 | 30% / 40% | Oral, flexible | 25mg | 250mg | 11-weeks | No |
| **13** | Hollander et al. (Eric Hollander et al., 2000) | 2000 | Biol Psychiatry | Double Blind RCT Cross-over design, 2 arms | fluvoxamine vs. placebo | 6/7 | 43.0 /  36.2 | 100% / 100% | Oral, fixed & cross-over | 50mg | 250mg | 8-weeks | Yes |
| **14** | Grant et al.(Grant et al., 2014) | 2014 | J Clin Psychiatry | Double Blind RCT Parallel design, 2 arms | NAC vs. placebo | 13/15 | N/A | N/A | Oral, clinical judgement | 1200mg | 3gr | 12-weeks | No |
| **15** | Hollander et al. (E Hollander, Pallanti, Allen, Sood, & Rossi, 2005) | 2005 | Am J Psychiatry | Double Blind RCT Parallel design, 2 arms | lithium vs placebo | 18/22 | 40.0 /  47.7 | 50% / 64.7% | Oral, flexible | 300mg sust. release | 1200mg sust. release | 10-weeks | Yes |
| **16** | Grant et al. (Grant, Driessens, & Chamberlain, 2024) | 2024 | Clinical Neuropharm | Double Blind RCT Parallel design, 2 arms | silymarin vs placebo | 17/26 | 48.2/ 50.5 | 41.2% / 72% | Oral, titration | 300mg | 600mg | 8-weeks | No |

## References

**Alho, H., Mäkelä, N., Isotalo, J., Toivonen, L., Ollikainen, J., & Castrén, S**. (2022). Intranasal as needed naloxone in the treatment of gambling disorder: A randomised controlled trial. *Addictive Behaviors*, *125*. https://doi.org/10.1016/J.ADDBEH.2021.107127

**Berlin, H. A., Braun, A., Simeon, D., Koran, L. M., Potenza, M. N., McElroy, S. L., … Hollander, E.** (2013). A double-blind, placebo-controlled trial of topiramate for pathological gambling. *The World Journal of Biological Psychiatry : The Official Journal of the World Federation of Societies of Biological Psychiatry*, *14*(2), 121–128. https://doi.org/10.3109/15622975.2011.560964

**Black, D. W., Arndt, S., Coryell, W. H., Argo, T., Forbush, K. T., Shaw, M. C., … Allen, J**. (2007). Bupropion in the treatment of pathological gambling: a randomized, double-blind, placebo-controlled, flexible-dose study. *Journal of Clinical Psychopharmacology*, *27*(2), 143–150. https://doi.org/10.1097/01.JCP.0000264985.25109.25

**de Brito, A. M. C., de Almeida Pinto, M. G., Bronstein, G., Carneiro, E., Faertes, D., Fukugawa, V., … Tavares, H**. (2017). Topiramate Combined with Cognitive Restructuring for the Treatment of Gambling Disorder: A Two-Center, Randomized, Double-Blind Clinical Trial. *Journal of Gambling Studies*, *33*(1), 249–263. https://doi.org/10.1007/S10899-016-9620-Z/METRICS

**Fong, T., Kalechstein, A., Bernhard, B., Rosenthal, R., & Rugle, L.** (2008). A double-blind, placebo-controlled trial of olanzapine for the treatment of video poker pathological gamblers. *Pharmacology, Biochemistry, and Behavior*, *89*(3), 298–303. https://doi.org/10.1016/J.PBB.2007.12.025

**Grant, J. E., Driessens, C., & Chamberlain, S. R.** (2024). Milk Thistle (Silymarin) Treatment of Adults with Gambling Disorder: A Double-Blind, Placebo-Controlled Trial. *Clinical Neuropharmacology*, *in press*. Retrieved from https://ichgcp.net/clinical-trials-registry/NCT02337634

**Grant, J. E., Kim, S. W., Hollander, E., & Potenza, M. N.** (2008). Predicting response to opiate antagonists and placebo in the treatment of pathological gambling. *Psychopharmacology*, *200*(4), 521–527. https://doi.org/10.1007/s00213-008-1235-3

**Grant, J. E., Kim, S. W., Potenza, M. N., Blanco, C., Ibanez, A., Stevens, L., … Zaninelli, R**. (2003). Paroxetine treatment of pathological gambling: a multi-centre randomized controlled trial. *International Clinical Psychopharmacology*, *18*(4), 243–249. https://doi.org/10.1097/00004850-200307000-00007

**Grant, J. E., Odlaug, B. L., Chamberlain, S. R., Potenza, M. N., Schreiber, L. R. N., Donahue, C. B., & Kim, S. W.** (2014). A randomized, placebo-controlled trial of N-acetylcysteine plus imaginal desensitization for nicotine-dependent pathological gamblers. *The Journal of Clinical Psychiatry*, *75*(1), 39–45. https://doi.org/10.4088/JCP.13M08411

**Grant, J. E., Potenza, M. N., Hollander, E., Cynningham-Williams, R., Nurminen, T., Smits, G., & Kallio, A**. (2006). Multicenter investigation of the opioid antagonist nalmefene in the treatment of pathological gambling. *The American Journal of Psychiatry*, *163*(2), 303–312. https://doi.org/10.1176/APPI.AJP.163.2.303

**Hollander, E, Pallanti, S., Allen, A., Sood, E., & Rossi, N.** (2005). Does sustained-release lithium reduce impulsive gambling and affective instability versus placebo in pathological gamblers with bipolar spectrum disorders? *The American Journal of Psychiatry*, *162*(1), 137–145. https://doi.org/10.1176/APPI.AJP.162.1.137

**Hollander, Eric, Decaria, C. M., Finkell, J. N., Begaz, T., Wong, C. M., & Cartwright, C.** (2000). A randomized double-blind fluvoxamine/placebo crossover trial in pathologic gambling. *Biological Psychiatry*, *47*(9), 813–817. https://doi.org/10.1016/S0006-3223(00)00241-9

**Kim, S. W., Grant, J. E., Adson, D. E., & Shin, Y. C.** (2001). Double-blind naltrexone and placebo comparison study in the treatment of pathological gambling. *Biological Psychiatry*, *49*(11), 914–921. https://doi.org/10.1016/S0006-3223(01)01079-4

**Kim, S. W., Grant, J. E., Adson, D. E., & Zaninelli, R.** (2002). A Double-Blind Placebo-Controlled Study of the Efficacy and Safety of Paroxetine in the Treatment of Pathological Gambling. *The Journal of Clinical Psychiatry*, *63*(6), 4776. Retrieved from https://www.psychiatrist.com/jcp/ocd/pathological-gambling/double-blind-placebo-controlled-study-efficacy-safety-paroxetine-pathological-gambling

**Kovanen, L., Basnet, S., Castrén, S., Pankakoski, M., Saarikoski, S. T., Partonen, T., … Lahti, T.** (2016). A Randomised, Double-Blind, Placebo-Controlled Trial of As-Needed Naltrexone in the Treatment of Pathological Gambling. *European Addiction Research*, *22*(2), 70–79. https://doi.org/10.1159/000435876

**McElroy, S. L., Nelson, E. B., Welge, J. A., Kaehler, L., & Keck, P. E**. (2008). Olanzapine in the treatment of pathological gambling: a negative randomized placebo-controlled trial. *The Journal of Clinical Psychiatry*, *69*(3), 433–440. https://doi.org/10.4088/JCP.V69N0314

## PRISMA-P

Preferred reporting items for systematic review and meta-analysis protocols) 2015 checklist:

| **Section and topic** | **Item No** | **Checklist item** |
| --- | --- | --- |
| **Administrative information** | | |
| Title: |  |  |
| Identification | 1a | Identify the report as a protocol of a systematic review *(identified as meta-analysis)* |
| Update | 1b | If the protocol is for an update of a previous systematic review, identify as such *(not applicable)* |
| Registration | 2 | If registered, provide the name of the registry (such as PROSPERO) and registration number *(PROSPERO registration provided)* |
| Authors: |  |  |
| Contact | 3a | Provide name, institutional affiliation, e-mail address of all protocol authors; provide physical mailing address of corresponding author *(in title page)* |
| Contributions | 3b | Describe contributions of protocol authors and identify the guarantor of the review *(see author contributions p20 line 434)* |
| Amendments | 4 | If the protocol represents an amendment of a previously completed or published protocol, identify as such and list changes; otherwise, state plan for documenting important protocol amendments *(not applicable)* |
| Support: |  |  |
| Sources | 5a | Indicate sources of financial or other support for the review *(see p18 line 407)* |
| Sponsor | 5b | Provide name for the review funder and/or sponsor  *(see p18 line 407)* |
| Role of sponsor or funder | 5c | Describe roles of funder(s), sponsor(s), and/or institution(s), if any, in developing the protocol  *(not applicable)* |
| **Introduction** | | |
| Rationale | 6 | Describe the rationale for the review in the context of what is already known *(see p5-6)* |
| Objectives | 7 | Provide an explicit statement of the question(s) the review will address with reference to participants, interventions, comparators, and outcomes (PICO) *(see p6-7)* |
| **Methods** | | |
| Eligibility criteria | 8 | Specify the study characteristics (such as PICO, study design, setting, time frame) and report characteristics (such as years considered, language, publication status) to be used as criteria for eligibility for the review *(see materials and methods lines 126-140)* |
| Information sources | 9 | Describe all intended information sources (such as electronic databases, contact with study authors, trial registers or other grey literature sources) with planned dates of coverage *(see materials and methods lines 118-125)* |
| Search strategy | 10 | Present draft of search strategy to be used for at least one electronic database, including planned limits, such that it could be repeated *(see materials and methods lines 118-125)* |
| Study records: |  |  |
| Data management | 11a | Describe the mechanism(s) that will be used to manage records and data throughout the review *(see materials and methods lines 132-154)* |
| Selection process | 11b | State the process that will be used for selecting studies (such as two independent reviewers) through each phase of the review (that is, screening, eligibility and inclusion in meta-analysis) *(see materials and methods line 132)* |
| Data collection process | 11c | Describe planned method of extracting data from reports (such as piloting forms, done independently, in duplicate), any processes for obtaining and confirming data from investigators *(see materials and methods line 132)* |
| Data items | 12 | List and define all variables for which data will be sought (such as PICO items, funding sources), any pre-planned data assumptions and simplifications *(see materials and methods line 132 and 133-154)* |
| Outcomes and prioritization | 13 | List and define all outcomes for which data will be sought, including prioritization of main and additional outcomes, with rationale *(see materials and methods line 132 and 133-154* |
| Risk of bias in individual studies | 14 | Describe anticipated methods for assessing risk of bias of individual studies, including whether this will be done at the outcome or study level, or both; state how this information will be used in data synthesis *(see materials and methods line 132 and 133-140)* |
| Data synthesis | 15a | Describe criteria under which study data will be quantitatively synthesised *(see materials and methods line 132 and 156-180)* |
|  | 15b | If data are appropriate for quantitative synthesis, describe planned summary measures, methods of handling data and methods of combining data from studies, including any planned exploration of consistency (such as I^2^, Kendall’s τ) *(see materials and methods line 132 and 156-180)* |
|  | 15c | Describe any proposed additional analyses (such as sensitivity or subgroup analyses, meta-regression) *(see materials and methods line 132 and 156-180)* |
|  | 15d | If quantitative synthesis is not appropriate, describe the type of summary planned *(not applicable)* |
| Meta-bias(es) | 16 | Specify any planned assessment of meta-bias(es) (such as publication bias across studies, selective reporting within studies) *(see materials and methods line 132 and 181-184)* |
| Confidence in cumulative evidence | 17 | Describe how the strength of the body of evidence will be assessed (such as GRADE) (*(see materials and methods line 132 and 181-184, also see limitations)* |
